# BTK inhibitors in the treatment of hematological malignancies and inflammatory diseases: mechanisms and clinical studies

**DOI:** 10.1186/s13045-022-01353-w

**Published:** 2022-10-01

**Authors:** Aqu Alu, Hong Lei, Xuejiao Han, Yuquan Wei, Xiawei Wei

**Affiliations:** Laboratory of Aging Research and Cancer Drug Target, State Key Laboratory of Biotherapy and Cancer Center, National Clinical Research Center for Geriatrics, West China Hospital, Sichuan University, Chengdu, 610041 China

**Keywords:** BTK, Inhibitors, Hematological malignancies, Inflammatory diseases, Signaling pathways, Clinical trials

## Abstract

Bruton’s tyrosine kinase (BTK) is an essential component of multiple signaling pathways that regulate B cell and myeloid cell proliferation, survival, and functions, making it a promising therapeutic target for various B cell malignancies and inflammatory diseases. Five small molecule inhibitors have shown remarkable efficacy and have been approved to treat different types of hematological cancers, including ibrutinib, acalabrutinib, zanubrutinib, tirabrutinib, and orelabrutinib. The first-in-class agent, ibrutinib, has created a new era of chemotherapy-free treatment of B cell malignancies. Ibrutinib is so popular and became the fourth top-selling cancer drug worldwide in 2021. To reduce the off-target effects and overcome the acquired resistance of ibrutinib, significant efforts have been made in developing highly selective second- and third-generation BTK inhibitors and various combination approaches. Over the past few years, BTK inhibitors have also been repurposed for the treatment of inflammatory diseases. Promising data have been obtained from preclinical and early-phase clinical studies. In this review, we summarized current progress in applying BTK inhibitors in the treatment of hematological malignancies and inflammatory disorders, highlighting available results from clinical studies.

## Background

Bruton’s tyrosine kinase (BTK) was firstly reported to be related to the inherited immunodeficiency disease x-linked agammaglobulinemia (XLA) in 1993, mutations of which cause a disorder in the transformation of pre-B cells in the bone marrow into mature peripheral B cells [1, 2]. At first, BTK was thought to be expressed only in B cells since no significant developmental and functional defects were observed in other immune cells of XLA patients. In consistent, a point mutation in the BTK gene led to the X-linked immunodeficiency (XID) phenotype in mice, which showed B cell-specific abnormality characterized by the inability to produce antibodies [3, 4]. Soon after, scientists discovered that stimulation of B cell receptors (BCR) can induce the tyrosine phosphorylation and activation of BTK in mature B cells [5–7]. BTK is also constitutively phosphorylated in pre-B cells and plays a functional role in pre-BCR signaling [7]. The pre-BCR is an immature form of BCR, which transduces signals for cell growth and differentiation [8]. Therefore, in XLA patients, defects in BTK’s function resulted in hampered pre-BCR signaling and B cell development.

**Fig. 1:**
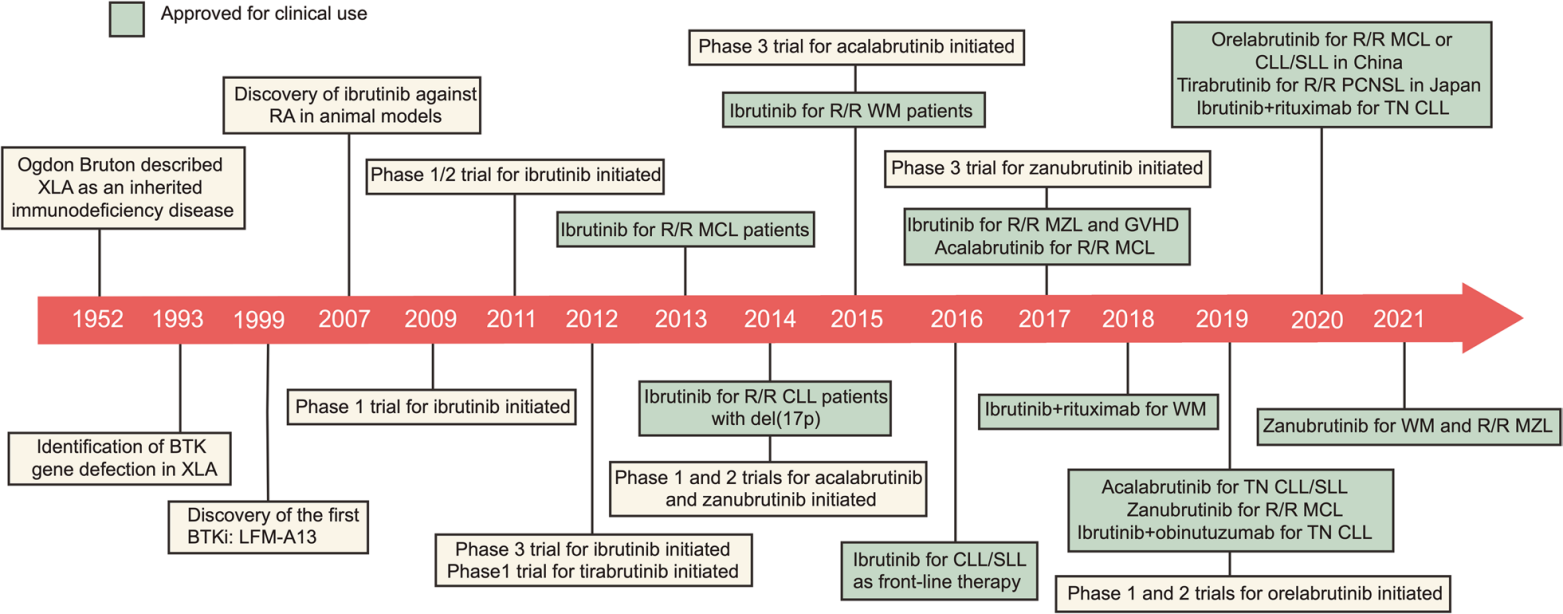
Key milestones in the development of BTK inhibitors, with approved indications. *XLA* X-linked agammaglobulinemia; *GVHD* graft-versus-host disease; *R/R* relapsed and refractory; *TN* treatment-naïve

Then, it is demonstrated that besides normal B cells, BTK is also expressed in malignant B cells [9, 10]. BTK is not only indispensable for B lineage development and function but inhibits Fas/CD95-induced apoptosis in lymphoid B cells [11, 12]. These results inspired the development of BTK inhibitors (BTKi) in treating B cell malignancies. In 1999, Mahajan et al. rationally designed the first BTKi named LFM-A13, which showed synergistic anti-leukemia effects with ceramide or vincristine in vitro [13]. After that, plenty of upgraded BTKi have been developed gradually, with higher efficacy and selectivity. Ibrutinib was the first-in-class BTKi that received its first approval by the U.S. Food and Drug Administration (FDA) in 2013 for the treatment of relapsed and refractory (R/R) mantle cell lymphoma (MCL). The approval of ibrutinib has an epoch-making significance since it offered the concept of chemotherapy-free treatment of hematological cancers. It is so popular that the global market size of ibrutinib grew to about 9.44 billion dollars in 2020 and was estimated to reach 66.28 billion dollars by 2030. It ranked fourth in the list of the top10 cancer drugs by sales in 2021. The success of ibrutinib promoted the exploration of second- and third-generation BTKi, aiming to reduce off-target toxicities and overcome acquired resistance, which is common in patients receiving continuous BTKi treatment. Among those inhibitors, acalabrutinib, zanubrutinib, tirabrutinib, and orelabrutinib have received accelerated or conditional approval for the treatment of multiple B cell malignancies (Fig. 1).

BTK is also expressed in many other hematopoietic cells, including macrophages, granulocytes, mast cells, osteoclasts, etc. [10, 14]. Meanwhile, BTK is involved in other signaling pathways, including Toll-like receptor (TLR) signaling, chemokine receptor signaling, and Fc receptor (FcR) signaling [15–17]. Recent studies revealed that BTK plays a significant role in the pathogenesis of inflammatory diseases, especially autoimmune diseases. Autoimmune disorders are characterized by a loss of self-tolerance, abnormal B cell activation, and subsequent generation of autoreactive antibodies [18]. Animal models indicated that BTK is essential for defining the threshold for B cell activation and counterselection of autoreactive B cells via BCR signaling [19]. Transgenic mice overexpressing BTK spontaneously formed systemic lupus erythematosus (SLE)-like autoimmune pathology involving multiple organs. BTK is also critical for the production of inflammatory cytokines in innate immune cells [20]. Thus, BTK overactivation may contribute to the development of chronic inflammation or acute hyperinflammatory status, making BTK a promising therapeutic target. Results from preclinical and clinical studies have supported BTKi’s activity in reducing inflammation and the production of autoantibodies [21, 22]. Since most patients with inflammatory diseases show far less severe symptoms than those with cancer, BTKi used for these patients must be highly selective and effective with minimal toxicities. Evobrutinib was the first BTKi reported to be used in the clinic in 2019 against a chronic inflammatory disorder: active relapsing–remitting multiple sclerosis (MS) [23]. The positive results of evobrutinib in MS patients have promoted the development and evaluation of BTKi in different kinds of inflammatory disorders.

Although some reviews have discussed BTKi before, most of them focused on limited disease types or agents and were outdated considering the fast pace of drug development both in laboratories and the clinic. In this review, we provided a state-of-the-art and comprehensive summary of the current status of BTKi in managing different kinds of B cell malignancies, including chronic lymphocytic leukemia (CLL)/small lymphocytic lymphoma (SLL), MCL, Waldenstrom macroglobulinemia (WM), marginal zone lymphoma (MZL), diffuse large B cell lymphoma (DLBCL), follicular lymphoma (FL), and multiple myeloma (MM). Since accumulating evidence indicated that BTKi is an emerging therapeutic strategy against inflammatory diseases [24, 25], we also discussed the application of BTKi in the management of inflammatory disorders, such as SLE, rheumatoid arthritis (RA), MS, and idiopathic thrombocytopenic purpura (ITP). Current accessible clinical trial data are highlighted. To help readers better understand how BTKi work, we also gave a brief introduction of BTK’s role in some key signaling pathways. We hope this article would help guide the clinical use and basic research of BTKi and inspire the exploration of novel inhibitors for scientists and pharmacologists.

## BTK signaling pathways

**Fig. 2:**
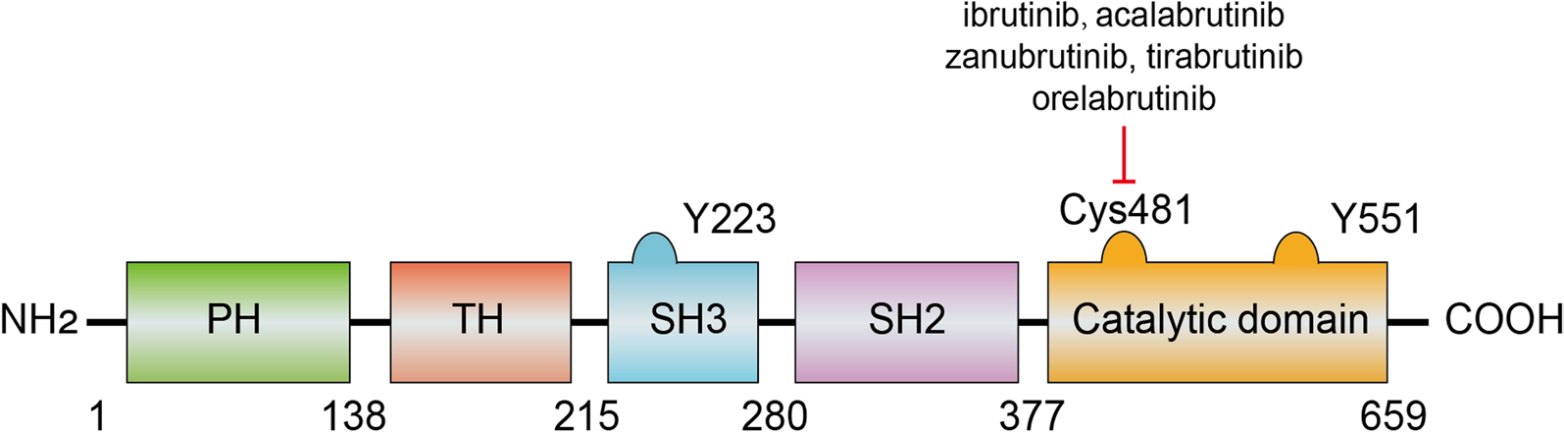
BTK’s structure and interactions. BTK contains 659 amino acids and five domains from the N-terminus to the C-terminus, including an amino-terminal pleckstrin homology domain (PH domain), a proline-rich TEC homology (TH) domain, the SRC homology domains SH2 and SH3 domains, and a catalytic domain. Phosphorylation of the Y551 and Y223 sites is necessary for the activation of BTK. Cys481 residues on the catalytic domain are the main targets for the approved BTK inhibitors

**Fig. 3:**
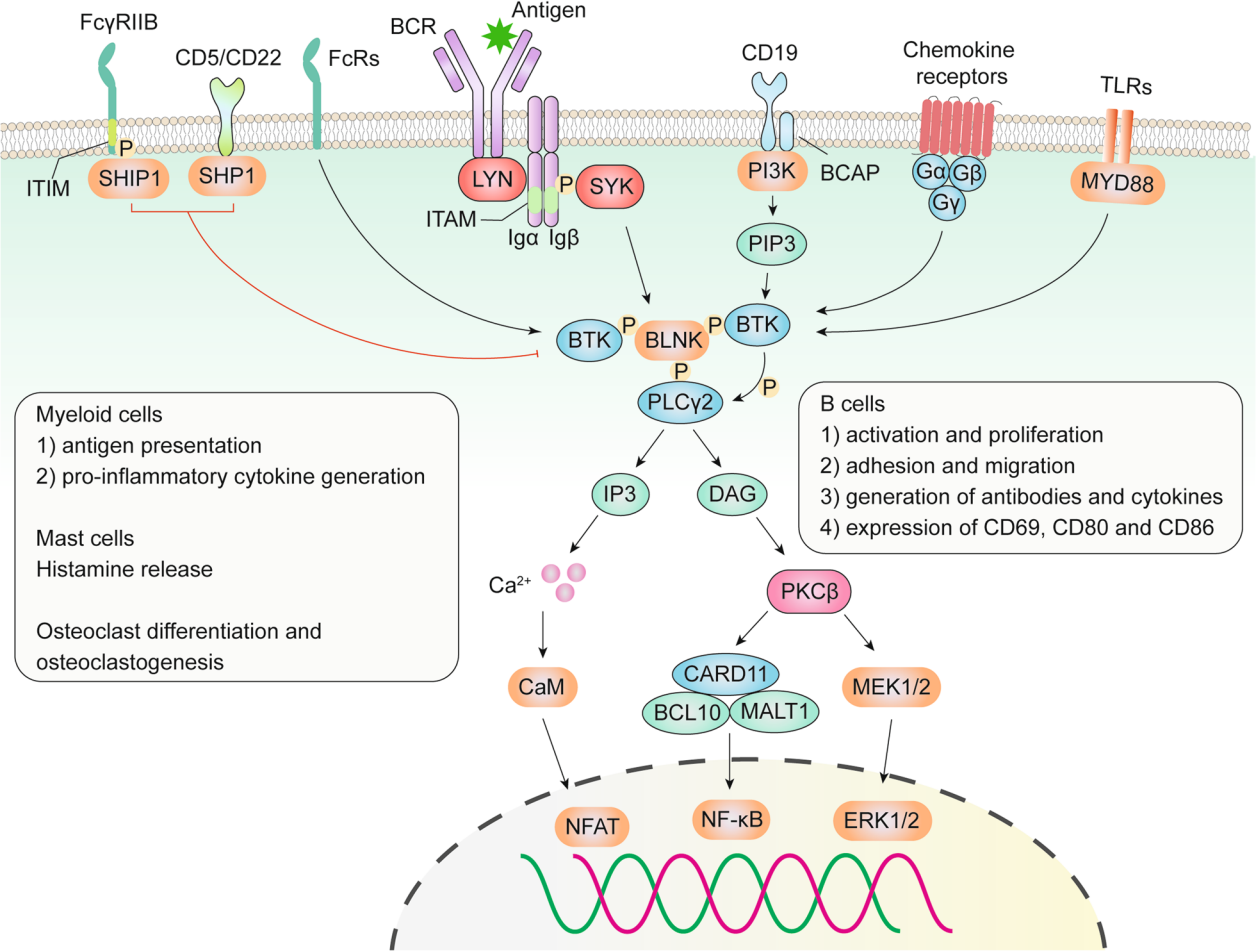
Role of BTK in BCR signaling, TLR signaling, chemokine receptor signaling, and FcR signaling pathways. Upon antigen binding, BCR signaling is activated involving the formation of a “micro-signalosomes” composed of PI3K, BTK, BLNK, and PLCγ2. Activated BTK leads to the phosphorylation of PLCγ2 and stimulates its lipase activity, resulting in Ca^2+^ influx and the activation of the NFAT transcription factors via calmodulin (CaM). Activation of PLCγ2 also induces the activation of PKCβ via DAG, which subsequently activated the ERK1/2 and NF-κB signaling pathways. Activation of BCR signaling can promote B cell proliferation, survival, and functions. In addition, activation of TLR and chemokine receptors can activate BTK and regulate the adhesion, migration, and production of pro-inflammatory cytokines in *B* cells and myeloid cells. BTK-dependent FcR signaling is essential for histamine release from mast cells, enhanced antigen presentation and cytokine generation from myeloid cells, and controls osteoclast differentiation and osteoclastogenesis. SHIP1 and SHP1 are negative regulators of BTK’s activity

BTK is a non-receptor tyrosine kinase of the TEC family, which is highly conserved throughout evolution [26]. It contains 659 amino acids and five domains from the N-terminus to the C-terminus, including the SRC homology domains SH2 and SH3, an amino-terminal pleckstrin homology domain (PH domain), a proline-rich TEC homology (TH) domain, and a catalytic domain (Fig. 2) [27, 28]. Generally, BTK is located in the cytoplasm and can be temporarily recruited to the cell membrane upon activation [27]. This process is mediated by the binding of the PH domain to phosphatidylinositol lipids on the membrane [e.g., phosphatidylinositol-3,4,5-trisphosphate (PIP3)]. After translocation, BTK can be activated with two steps: (1) phosphorylation of BTK at the Y551 sites in the kinase domain by spleen tyrosine kinase (SYK) or SRC family kinase; (2) autophosphorylation of the Y223 sites in the SH3 domain as a result of Y551 phosphorylation, which can fully stimulate the kinase activity of BTK and stabilize its active conformation [29]. The TH domain contains a zinc-finger motif which is essential for the optimal activity and stability of BTK [27, 28]. BTK is expressed in a plethora of hematopoietic cells, including B cells, macrophages, neutrophils, mast cells, eosinophils, and platelets [10, 14]. Activation of BTK participates in various signaling pathways, including BCR signaling, chemokine receptor signaling, TLR signaling, and FcR signaling (Fig. 3).

### BCR signaling

The BCR is composed of a transmembrane immunoglobulin complex with antigen-binding sites on the cell surface. Activation of BCR signaling is critical for B cell development and function. B cells originate in bone marrow, where rearrangement of immunoglobulin *V*, *D*, and *J* segments results in unique BCR. Only B cells that express a functional BCR and pass the negative selection for autoreactivity can survive. Subsequently, when stimulated by various antigens in secondary lymphoid organs, mature B cells will go through somatic hypermutations and only these express high-affinity BCRs will be positively selected, leading to B cell expansion, diversification and differentiation into memory B cells or antibody-secreting plasma cells. The BCR signaling can be activated in two different pathways: antigen-dependent signaling and antigen-independent tonic signaling. The tonic BCR signaling promotes B cell survival via the PI3K-AKT-mTOR pathway, which is very common in Burkitt lymphoma and germinal center B-cell-like DLBCL (GCB‑DLBCL) [30]. Since the tonic BCR signaling pathway doesn’t involve BTK, it will not be discussed in detail here. BCR can also be activated by antigen binding, which induces a downstream cascade primarily mediated by protein kinase phosphorylation. Different forms of antigens can be recognized by BCR, including soluble antigens in the lymphatic fluid or surface antigens presented by antigen-presenting cells (e.g., macrophages and follicular dendritic cells) [31]. In pathological conditions, continuous antigen stimulation from microorganisms or autoantigens can cause overactivation of BCR signaling, leading to the development and proliferation of malignant B cells or the generation of excessive autoreactive antibodies and pro-inflammatory cytokines. For instance, chronic *Helicobacter pylori* infection has been reported to be associated with the development of gastric mucosa-associated lymphoid tissue (MALT) lymphoma [32]. Autoantigens exposed to apoptotic cells can also activate BCR signaling and drive the pathogenesis of B cell cancers like CLL and activated B cell (ABC)-DLBCL [33–36].

The BCR complex is non-covalently coupled with the Igα/Igβ (also known as CD79a/b) heterodimer. When stimulated by antigen binding, the SRC family (most probably LYN) can phosphorylate the immunoreceptor tyrosine-based activation motifs (ITAMs) on Igα and Igβ, creating docking sites for the activation of SYK [37]. Activated SYK can further recruit and phosphorylate B cell linker protein (BLNK, also known as SLP65 and BASH) to provide a scaffold for the recruitment and phosphorylation of various signaling molecules, including SYK, BTK, and phospholipase C-γ2 (PLCγ2) [38, 39]. Simultaneously, LYN phosphorylates tyrosine residues in the cytoplasmic tail of CD19 (a co-receptor of BCR), which can bind and activate phosphoinositide 3-kinase (PI3K) and VAV [40, 41]. Activation of SYK and BTK also mediates the tyrosine phosphorylation of the B cell adapter for PI3K (BCAP) which can recruit PI3K [42]. Then, PI3K phosphorylates PIP2 to produce PIP3, attracting BTK to cell membranes and allowing SYK and LYN to fully activate BTK. SH2-domain-containing inositol polyphosphate 5’phosphatase-1 (SHIP1) is a negative regulator of BTK activation, whose activity is dependent on LYN-mediated phosphorylation of immune tyrosine inhibitory motifs (ITIMs) on FcγRIIB [29, 43]. SHIP1 catalyzes the dephosphorylation of PIP3 to reduce BTK’s membrane association. SH2 domain-containing protein tyrosine phosphatase-1 (SHP1) is another inhibitory molecule downstream of CD5 and CD22. It can directly dephosphorylate tyrosine on BTK [44].

The recruitment of BTK, PLCγ2, VAV, and PI3K to BLNK promotes the formation of highly coordinated “micro-signalosomes”, where BTK phosphorylates PLCγ2 and stimulates its lipase activity [45, 46]. Activated PLCγ2 cleaves PIP2 into two second messengers: inositol triphosphate (IP3) and diacylglycerol (DAG). IP3 is essential for regulating intracellular Ca^2+^ homeostasis, thus activating the nuclear factor of activated T cells (NFAT) transcription factors via calmodulin (CaM). DAG can activate protein kinase Cβ (PKCβ), which subsequently induces RAS signaling-dependent phosphorylation of ERK1/2. PKCβ also activates the NF-κB pathway involving a scaffold complex with caspase recruitment domain-containing protein 11 (CARD11), BCL-10, and mucosa-associated lymphoid tissue lymphoma translocation protein 1 (MALT1). Activation of BCR signaling pathways promotes B cell activation and proliferation, increases the generation of antibodies and cytokines, and elevates the expression of co-stimulatory molecules (CD69, CD80, and CD86) on B cells [27, 28, 47].

### TLR signaling

BTK is also involved in TLR signaling in an MYD88-dependent manner. Upon stimulation with damage/pathogen-associated molecular patterns (DAMP/PAMP), MYD88 would be recruited to most TLRs (excluding TLR3 and some TLR4) [27, 28]. BTK can be activated by directly interacting with the intercellular domains of TLRs, MYD88, MYD88 adaptor-like (Mal) protein, IL-1R-associated kinase 1 (IRAK1), and TIR-domain-containing adapter-inducing interferon-β (TRIF), further activating downstream transcription factors including NF-κB, activator protein 1 (AP1) and interferon regulatory factor (IRF3) to promotes cell proliferation and functions [27, 28]. CD38 can inhibit endotoxin-triggered TLR4 signaling by inhibiting the activation of BTK [48]. This is mediated by the activation of SHP2 that can dephosphorylate BTK, thus preventing the downstream signaling pathways involving NF-κB and NLRP3 in macrophages. Interestingly, BTK may be an essential factor in the interconnection between BCR signaling and TLR signaling. Kenny et al. reported that BTK is necessary for the colocalization of TLR9 and BCR in an autophagosome-like compartment. This promotes the synergistic effects of the different signaling pathways on IL-6 production and up-regulation of surface maturation markers in B cells [49].

### Chemokine receptor signaling

Chemokine receptors are G-protein coupled receptors and are composed of *α*, *β*, and *γ* subunits (G*α*, G*β*, and G*γ*) [50]. BTK is essential for CXCL12/CXCR4 and CXCL13/CXCR5 signaling pathways that regulate cell adhesion and migration [51, 52]. Upon binding to CXCL12/13, G*α* and G*βγ* subunits can activate BTK by binding to its PH and TH domains. Moreover, G*βγ* subunits can bind to the catalytic domain and stimulate PIP3-dependent membrane anchorage [27, 53]. BTK may also be involved in CCL19/CCR7 mediated signaling since the BTKi, PCI-32765, inhibited CCL19-induced adhesion and migration of primary CLL lines [17].

### FcR signaling pathway

BTK is an important component of FcR signaling pathways, including FcεR and FcγR. Activation of FcεR increases histamine release from mast cells, while FcγR activation enhances the antigen presentation and the generation of pro-inflammatory cytokines from myeloid cells [22, 54]. BTK-dependent FcR signaling is also required for RANKL (receptor activator of NF-κΒ ligand)-controlled osteoclast differentiation and osteoclastogenesis [55, 56]. Therefore, overactivation of BTK plays a vital role in the pathogenesis of systemic inflammatory diseases.

## An overview of BTK inhibitors

**Fig. 4:**
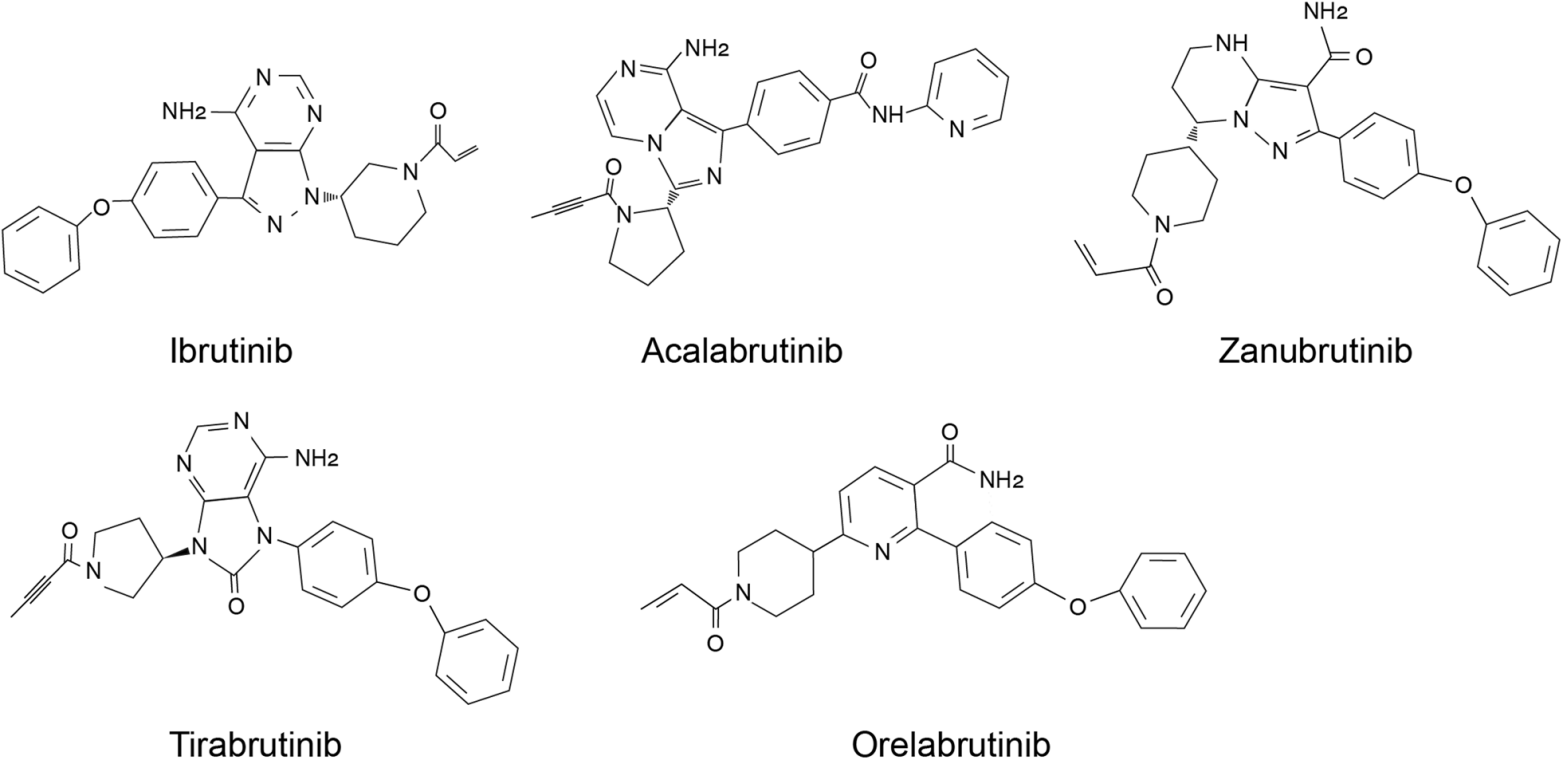
Chemical structures of the approved BTK inhibitors

In recent decades, the development of BTKi has made a great contribution to the management of hematological malignancies and inflammatory disorders. BTKi can be divided into covalent and non-covalent inhibitors according to different acting mechanisms. Covalent inhibitors bind to the wild-type (WT) or mutant cysteine 481 (Cys481) residue via covalent bonding. Non-covalent inhibitors can occupy the ATP binding pocket or a specific H3 pocket of BTK via non-covalent forces like hydrogen bonding or hydrophobic interactions [57]. All the currently approved agents belong to irreversible covalent inhibitors that could potently and persistently inhibit the enzyme activity of BTK (Fig. 4) [58]. Owing to the off-target effects and the emergence of mutants in the BTK binding sites, unwanted side effects and acquired resistance may display in patients receiving BTKi. To address these obstacles, pharmaceutical companies and academic institutions are developing novel BTKi with higher selectivity and broader binding site coverage. In this section, a brief introduction of BTKi will be provided, highlighting their cons and pros when used in humans.

### Clinically approved BTK inhibitors

Five BTKi have been approved for use in humans, including ibrutinib, acalabrutinib, zanubrutinib, tirabrutinib, and orelabrutinib. Ibrutinib, also named PCI-32765, is the first-generation BTKi and was discovered in 2007. Since 2013, it has been intermittently approved for the treatment of MCL, CLL/SLL, chronic graft-versus-host disease (GVHD), WM, and MZL as monotherapy or combined therapy (Table 1) [59–61]. The triumph of ibrutinib represents a milestone since it brought about the possibility of chemotherapy-free management of B cell malignancies. However, ibrutinib can also inhibit other kinases, including but not limited to the epidermal growth factor receptor (EGFR) family kinases, SRC family kinases, TEC-family kinases, etc. [62, 63]. These nonspecific bindings are caused by the equivalent cysteine residues in the active sites of other kinases [64], bringing about the possibility of off-target activity and treatment-related adverse effects (AEs). Serious AEs may lead to treatment discontinuation, limiting its wide application. Therefore, it is important to develop BTKi with higher selectivity and fewer off-target toxicities.

**Table 1:** A summary of approved BTK inhibitors and those under clinical trials

Inhibitor	Binding mechanism	IC_50_ (nM)	Selectivity	Administration	Status	Refs.
*Approved*						
Ibrutinib	First-generation, irreversible, covalent binding to Cys481	0.5	Moderate	420/560/840 mg, QD	Approved for: CLL, MCL, GVHD, WM, and MZL Phase 3: AML Phase 2: DLBCL, HCL, CNSL, MM, wAIHA, COVID-19, FL, RS, and ALL Phase 1: R/R T cell lymphoma	[28, 57]
Acalabrutinib	Second-generation, irreversible, covalent binding to Cys481	3.0–5.1	High	100 mg BID	Approved for: CLL and R/R MCL Phase 3: DLBCL and COVID-19 Phase 2: WM, CNSL, wAIHA, FL, RS, and RA Phase 1: MZL, MM, and AML	[28, 57]
Zanubrutinib		0.3	High	160 mg BID, or 320 mg QD	Approved for: R/R MCL; WM, and R/R MZL Phase 3: hemophagocytic lymphohistiocytosis, CLL, and DLBCL Phase 2: NMOSD, ITP, RS, SLE, COVID-19, and CNSL Phase 1: AML	[65]
Orelabrutinib		1.6	High	150 mg QD	Conditionally approved for R/R MCL and R/R CLL in China Phase 3: PCNSL, DLBCL, and SLE Phase 2: ITP, RMS, and FL	[66]
Tirabrutinib		6.8	High	480 mg QD	Approved for R/R PCNSL in Japan Phase 2: Pemphigus, CLL, SS, WM, MCL, and MZL Phase 1: RA	[67]
*Under clinical trials*						
Spebrutinib (CC-292)	Second-generation, irreversible, covalent binding to Cys481	< 0.5	High	1000 mg QD, or 500 mg BID	Phase 2: acute RA Phase 1: DLBCL and FL	[68, 69]
Branebrutinib (BMS-986195)		0.1	High	1–10 mg QD	Phase 2: atopic dermatitis, RA, SLE, and SS	[55, 70]
SHR-1459		3	High	300 mg QD	Phase 2: R/R B cell NHL and PMN	[71]
DTRMWXHS-12		0.7	High	200 mg QD	Phase 2: R/R CLL and R/R NHL Phase 1: MCL	[72]
Tolebrutinib (SAR 442,168)		0.7	High	60 mg QD	Phase 3: MS and Myasthenia Gravis	[73, 74]
Evobrutinib (M2951)		38–58	Moderate	75 mg QD, or 75 mg BID	Phase 3: RMS Phase 2: SLE and RA	[74, 75]
Elsubrutinib (ABBV-105)		0.18	High		Phase 2: SLE and RA	[76]
AC0058TA		–	High	50/100/200 mg QD, or 100 mg BID	Phase 1: SLE	[77]
TG-1701		6.7	High	–	Phase 1: CLL and NHL	[78]
M7583		1.48	High	900 mg QD, or 300 mg BID	Phase 2: B cell malignancies	[79, 80]
Nemtabrutinib (ARQ 531, MK-1026)	Third-generation, reversible, non-covalent, binding to both WT BTK and BTK^Cys481S^ mutant	MT: 0.85 Mut: 0.39	Moderate	65 mg QD	Phase 2: CLL/SLL, RS, MZL, MCL, FL, and WM	[57, 81, 82]
Pirtobrutinib (LOXO-305)		0.85	High	200 mg	Phase 3: CLL/SLL and MCL Phase 2: NHL	[83]
Fenebrutinib (GDC-0853)		WT: 0.9 Mut: 1.6	High	50/150 mg QD, or 200 mg BID	Phase 3: RMS Phase 2: CSU, SLE, RA Phase 1: CLL and DLBCL	[84, 85]
Vecabrutinib (SNS-062)		WT: 4.6 Mut 1.1	High	25 mg escalated to 500 mg	Phase 2: B lymphoid cancers	[86, 87]
HMPL-760		–	High	–	Phase 1: CLL/SLL and NHL	
BMS-986142	Non-covalent, reversible binding to BTK	0.5	High		Phase 2: RA and SS	[88]
BIIB091		0.071	High	–	Phase 1: healthy volunteers	[89, 90]
Rilzabrutinib (PRN1008)	Third-generation, reversible, transient covalent binding to Cys481	1.3	High	400 mg BID	Phase 3: ITP Phase 2: wAIHA, asthma, atopic dermatitis, CSU, IgG4-related disease, and Pemphigus	[54, 91]
PRN473		1.8	High	Multiple topical doses	Phase 2: atopic dermatitis	[54]
SN-1011		–	High	–	Phase 1: healthy volunteers	
Remibrutinib (LOU064)	Covalent binding to an inactive conformation of BTK	1.3	High	100 mg QD	Phase 3: RMS, CSU Phase 2: asthma, SS, hidradenitis suppurativa	[92, 93]
NX-2127	Catalyze ubiquitylation and proteasomal degradation of BTK and BTK^Cys481S^ mutant	< 5	–	100–300 mg QD	Phase 1: B cell malignancies	[94]

*QD* once daily; *BID* twice daily; *WT* wild type; *Mut* mutant; *GVHD* graft-versus-host disease; *AML* acute myelocytic leukemia; *HCL* hairy cell leukemia; *CNSL* central nervous system lymphoma; *wAIHA* warm autoimmune hemolytic anemia; *RS* Richter’s syndrome; *ALL* acute lymphocytic leukemia; *NMOSD* neuromyelitis optica spectrum disorders; *SS* Sjögren's syndrome; *(R)MS* (relapsing) multiple sclerosis; *CSU* chronic spontaneous urticaria

The second-generation BTKi are designed to maximize the effects and selective BTK occupancy with reduced activity against off-target kinases. Representatives of these inhibitors include acalabrutinib (ACP-196), zanubrutinib (BGB-3111), tirabrutinib (ONO/GS-4059), and orelabrutinib (ICP-022). For instance, in vitro studies indicated that acalabrutinib exhibits higher target specificity than ibrutinib with 323-, 94-, 19-, and ninefold selectivity against other kinase members ITK, TXK, BMX, and TEC, respectively [95]. In comparison to ibrutinib, acalabrutinib treatment demonstrated fewer AEs in previously treated CLL patients, including atrial fibrillation (9.4% vs. 16.0%) and hypertension (9.4% vs. 23.3%) especially [96]. Acalabrutinib treatment led to a fivefold decline in AE-related treatment discontinuation than ibrutinib. Similarly, data from a phase III study reported that zanubrutinib treatment was related to better response and lower toxicity in WM patients than ibrutinib [97]. Orelabrutinib (ICP-022) showed excellent safety profiles and tolerability of orelabrutinib after long-term administration in humans [98]. In 2020, orelabrutinib received conditional approval in China for treating MCL and CLL/SLL patients who have received at least one prior therapy before [66]. However, the selectivity of tirabrutinib is about 2.4-fold lower than that of ibrutinib against the TEC kinase [99]. The simultaneous occupancy of BTK and TEC by tirabrutinib significantly inhibited osteoclast differentiation and bone loss driven by macrophage colony-stimulating factor (M-CSF) and RANKL [100]. Tirabrutinib received its first approval in Japan against R/R primary central nervous system lymphoma (PCNSL) in 2020.

### BTK inhibitors under clinical investigation

Spebrutinib (CC-292) belongs to the second-generation inhibitors that irreversibly and covalently bind to BTK^Cys481^ residues [71]. It is highly selective for BTK and has no impact on the SRC family kinases [69]. Spebrutinib can not only inhibit the proliferation of B cells in peripheral blood, but also reduce the generation of inflammatory chemokines (e.g., CXCL13) and cytokines (e.g., macrophage inflammatory protein 1β (MIP-1β), interleukin-6 (IL-6), IL-8, and tumor necrosis factor *α* (TNF-α)) [69, 101]. Branebrutinib (BMS-986195) is another covalent BTKi optimized from a reversible BTKi (BMS-986142) [55, 74]. It provides immediate occupancy and inactivation of BTK in vivo with a single 10 mg dose [70], supporting the application of a very low projected dosage in humans. Other representatives of second-generation BTKi include SHR-1459, DTRMWXHS-12 [72], tolebrutinib, evobrutinib, M7583 [79, 80], TG-1701 [78], elsubrutinib [76], and AC0058TA [77]. Among these inhibitors, tolebrutinib and evobrutinib can penetrate the blood–brain barrier, making them ideal options for central nervous system (CNS) disorders like MS [73, 75].

The second-generation BTKi are optimized to be more selective. However, they can still cause some noteworthy AEs. For instance, acalabrutinib can easily cause headaches and coughs, whereas zanubrutinib could cause a high frequency of neutropenia [96, 97]. Therefore, doctors should choose the appropriate inhibitor for different patients according to the diverse safety profiles. Moreover, the covalency and irreversibility that enhance the therapeutic effects are largely dependent on the conserved conformation at the binding sites. Mutations at the BTK^Cys481^ residues significantly compromise the efficacy and caused resistance to the irreversible BTKi [102]. Moreover, the irreversible binding still poses a selectivity risk since about 10 other human kinases contain equivalent Cys residues in the active sites [64]. Therefore, there is a great interest in developing third-generation BTKi with novel binding mechanisms.

Nemtabrutinib (ARQ 531, MK-1026), pirtobrutinib (LOXO-305), and vecabrutinib (SNS-062) are orally administered reversible and non-covalent inhibitors of both WT BTK and ibrutinib-resistant BTK^Cys481S^ mutant [81, 87, 103]. Nemtabrutinib has additional inhibitory effects on SRC, TEC, and TRK kinases, enhancing global inhibition of signaling pathways and increasing potency [57, 81]. In vivo, nemtabrutinib provided superior antitumor activity in CLL, DLBCL, acute myelocytic leukemia (AML), and Richter’s syndrome models compared to ibrutinib [81, 104]. Phase I study indicated that nemtabrutinib has a manageable safety profile and excellent antitumor activity as monotherapy in heavy R/R B cell malignancies pretreated with covalent BTKi [82]. The highly selective pirtobrutinib shows remarkable antitumor effects in the most aggressive ibrutinib–venetoclax–CAR-T triple-resistant MCL in a xenograft mouse model [103, 105]. In phase 1/2 study, pirtobrutinib was well tolerable and effective in CLL and SLL, including patients previously treated with covalent BTKi [83]. Another BTKi, vecabrutinib is highly selective and shows similar inhibitory activity on WT BTK to ibrutinib [87]. However, released data from a phase 1b study indicated that vecabrutinib is not as efficacious as expected in patients with advanced B lymphoid cancers, terminating further investigation of vecabrutinib on BTK-resistant CLL [86]. BMS-986142 is another reversible non-covalent BTKi designed for treating autoimmune diseases [88]. It demonstrated potent efficacy against RA, including collagen-induced arthritis (CIA) and collagen antibody-induced arthritis (CAIA) in mice [106]. Some other reversible BTKi like BIIB091, HMPL-760, and SN-1011 also showed desirable safety profiles in early-phase studies [89, 90].

Rilzabrutinib (PRN1008) and PRN473 are reversible covalent inhibitors of BTK^Cys481^ residues. This unique mechanism allows rapid dissociation of the small molecules from common triols while retaining persistent inhibition of BTK even after washout [54]. Rilzabrutinib inhibited the inflammatory functions of multiple immune cells without causing cell death. Additionally, rilzabrutinib reduced autoantibody-mediated FcγR and inhibits IgE-mediated, FcεR-dependent immune mechanisms in human basophils and mast cells [107]. These mechanisms explained the strong and sustained potency of rilzabrutinib in rodent arthritis and canine pemphigus models [54, 107]. Rilzabrutinib is associated with low-level and transient AEs and displays rapid and durable clinical activity in patients with ITP [91]. However, rilzabrutinib shows limited efficacy in pemphigus patients, resulting in the termination of a phase 3 study (NCT03762265).

The requirement of highly selective BTKi promoted the investigation of novel binding mechanisms for BTK. A significant advance in this field is the discovery of CGI1746, a reversible and ATP-competitive BTKi [22]. CGI1746 binds to and occupies the “H3” pocket within the un-phosphorylated BTK, inhibiting both the auto- and trans-phosphorylation of Tyr551, thus stabilizing BTK in its inactive conformation. Given the sequence specificity around the binding pocket, CGI1746 shows over 1000-fold selectivity of BTK than other kinases of the TEC and SRC families. CGI1746 demonstrated remarkable efficacy in animal models of inflammatory arthritis via preventing the production of antibodies and inhibiting pro-inflammatory cell infiltration and erosion of bone and cartilage [22], making CGI1746 a promising molecule for the treatment of inflammatory diseases. Fenebrutinib (GDC-0853) is optimized from CGI1746, which inhibits both natural and Cys481S-mutated BTK [84, 85]. The administration of fenebrutinib is highly tolerable and safe in early clinical trials of autoimmune diseases [84, 108]. Similar to CGI1746, remibrutinib (LOU064) covalently binds to a modified, inactive conformation of BTK, providing excellent kinase selectivity and strong potency (IC_50_ = 1.3 nM). Remibrutinib was shown to be the best-in-class BTKi designed to treat inflammatory diseases [92, 93]. A phase I clinical trial indicated that remibrutinib is well tolerated in healthy volunteers without any dose-limited toxicity [109]. Unlike cancer, there is a strong emphasis on treatment safety in inflammatory diseases, making the highly tolerable third-generation BTKi appealing choices.

NX-2127 is a unique inhibitor that prevents the functions of BTK by catalyzing the ubiquitylation and proteasomal degradation of BTK rather than via direct binding. It degraded 50% of cellular BTK at doses less than 5 nM. NX2127 can degrade both WT and mutant Cys481 binding sites, thus inducing substantially higher inhibition of the proliferation of BTK^Cys481^ mutant cell lines than ibrutinib. Mice receiving oral NX-2127 treatment showed superior tumor growth suppression than ibrutinib [94]. When given at doses of 200 mg daily, NX2127 achieved over 90% degradation of BTK in R/R B cell malignancies patients with mutations in the BTK gene [110].

## BTK inhibitors in hematological malignancies

### BTK inhibitors in CLL/SLL

CLL is the most common leukemia in the western world. It is featured by the clonal proliferation and accumulation of CD5^+^ mature B cells in the bone marrow, peripheral blood, and lymphoid organs [111]. CLL and SLL are diverse clinical presentations of the same pathological disease and will be collectively referred to as CLL in this article [112]. BTK is uniformly overexpressed and constitutively phosphorylated in CLL [113]. BTK signaling pathways substantially promoted the initiation, expansion, and migration of CLL cells. BTK deficiency and ibrutinib treatment abrogated or delayed tumor formation in a mice model of spontaneous CLL development. In contrast, BTK overexpression accelerated leukemia formation and mortality [114, 115]. Inhibiting BTK in primary CLL cells with ibrutinib or small interfering RNA (siRNA) promoted cell apoptosis and inhibited proliferation [114]. Oral administration of ibrutinib significantly inhibited cell signaling, induced cell death, and abrogated cell homing and adhesion in patients [116, 117].

BCR signaling pathway is also essential for the interaction of CLL cells with the tumor microenvironment. BTK inhibition can interfere with the survival signals in the microenvironment and increase the antitumor immunity [113, 118, 119]. For instance, ibrutinib treatment inhibited CXCL13 secretion, reduced CLL cell recruitment, and disaggregated CLL cell–macrophage interactions in the bone marrow microenvironment [119]. Inhibition of BTK signaling also led to enhanced humoral and cellular immunity in CLL patients. The former is characterized by the recovery of normal B cell numbers in peripheral blood, increased serum IgA levels and B cell precursors in the bone marrow [120]. The latter is caused by the improvement in T cell numbers and functions. Ibrutinib treatment elevated overall T cell numbers and reduced Treg/CD4^+^ T cell ratios. Moreover, inhibition of BTK downregulated the expression of immunosuppressive molecules, including programmed cell death protein 1 (PD-1), cytotoxic T lymphocyte-associated antigen 4 (CTLA-4), and CD200. This induced significantly elevated T cells expansion, activation, differentiation, and cytotoxicity against malignant CLL cells [118, 119, 121], promoting the combination of BTKi with immunotherapy in the management of CLL patients.

#### Ibrutinib

Ibrutinib is the firstly licensed BTKi for CLL. It has been approved for the treatment of multiple subgroups, including newly diagnosed CLL, R/R CLL, and elderly CLL patients, irrespective of high-risk gene lesions (TP53 mutation, IGHV mutation, and del(17p)) [122]. The approval of ibrutinib is based on promising data from randomized clinical trials. Early clinical trials indicated that ibrutinib treatment showed an overall response rate (ORR) of up to 71% with manageable AEs in R/R and previously untreated CLL patients, including those carrying del(17p), del(11q), unmutated IGHV status, and TP53 aberrations [123–129]. Phase 3 studies demonstrated that ibrutinib was much more effective than conventional therapies in managing CLL, including anti-CD20 antibodies (ofatumumab and rituximab), chlorambucil, and chemoimmunotherapy (CIT) regimes, as characterized by increased ORR, prolonged progress-free survival (PFS) and overall survival (OS) (Table 2) [130–138]. Long-term follow-up revealed that the ORR of ibrutinib remained relatively high after six years of continuous treatment, with the complete response (CR) rate increasing over time [129, 139]. Survival outcomes were robust in treatment-naïve (TN) patients (5-year PFS, 92%), but less favorable for R/R patients (5-year PFS, 44%), especially for those with del(17p) (5-year PFS, 26%). As for the untreated early-stage, asymptomatic CLL patients, the current strategy in clinical practice is to “watch and wait” since chemotherapy-based interventions showed no benefits in prolonging survival. In phase 3 CLL12 trial, ibrutinib promoted event-free survival without increasing overall toxicity in early-stage CLL patients [30]. Therefore, it is worthwhile to consider changing the current observational strategy to early ibrutinib therapy for CLL to prevent the risk of progression.

**Table 2:** Vital clinical trial data for the application of BTK inhibitors in hematological malignancies

Disease	Treatment	Phase	N	PFS*	OS (%)*	CR or MRR (%)	ORR (%)	Median follow-up (months)	Trial name	Grade ≥ 3 AEs (%)	NCT and Refs.
CLL	Ibrutinib	2	132	TN: 60 (92%) R/R: 60 (44%)	TN: 60 (92%) R/R: 60 (60%)	TN: 29% R/R: 10%	89%	60	PCYC-1102/1103	–	NCT01105247, NCT01109069 [139]
R/R CLL	Ibrutinib	2	144	24 (63%)	24 (75%)	10%	83%	27.6	RESONATE-17	–	NCT01744691 [126]
TN CLL	Ibrutinib	3	363	47.8 months	–	–	–	31	CLL12	–	NCT02863718 [140]
R/R CLL	a. Ibrutinib b. Rituximab	3	160	8.3 (74% vs. 11.9%)	NR vs. 26.1 months	3.8% vs. 0%	53.8% vs. 7.4%	17.8	–	82.7% vs. 59.6%	NCT01973387 [136]
TN CLL	a. Ibrutinib b. Chlorambucil	3	267	18 (90% *vs.* 52%)	24 (98% *vs.* 85%)	4% *vs*. 2%	86% *vs.* 35%	18.4	RESONATE-2	–	NCT01722487 [133]
R/R CLL	a. Ibrutinib b. Ofatumumab	3	391	44.1 vs. 8.1 months	67.7 vs. 65.1 months	11% (a)	91% (a)	65.3	RESONATE	57% vs. 47%	NCT01578707 [129, 130]
CLL	a. IR b. FCR	3	529	36 (89.4% vs. 72.9%)	36 (98.8% vs. 91.5%)	17.2% vs. 30.3%	95.8% vs. 81.1%	70	E1912	73.0% vs. 83.5%	NCT02048813 [137, 141]
TN CLL	a. BR b. Ibrutinib c. IR	3	547	24 (74% vs. 87% vs. 87%)	24 (95% vs. 90% vs. 94%)	26% vs. 7% vs. 12%	81% vs. 93% vs. 94%	38	–	Hematologic: 61% vs. 41% vs. 39% Non-hematologic: 63% vs. 74% vs. 74%	NCT01886872 [138]
TN CLL	IV	2	164	30 (≥ 95%)	–	46%	97%	31.3	CAPTIVATE	–	NCT02910583 [142]
TN CLL	IV	2	80	36 (93%)	36 (96%)	75% of uMRD	–	38.5		60%	NCT02756897 [143, 144]
R/R CLL	IV	2	230	–	–	53%	83%	–	VISION	57%	NCT03226301 [145]
R/R CLL	IV	2	53	21.1 (98%)	21.1 (100%)	51%	89%	21.1	CLARITY	–	EudraCT 2015–003,422-14 ISCRTN13751862[146]
CLL	IV + obinutuzumab	2	50	NR	NR	28%	TN: 84% R/R: 88%	TN: 24.2 R/R: 21.5	–	66%	NCT02427451 [147]
R/R CLL	a. Ibrutinib + ublituximab b. ibrutinib	3	224	NR vs. 35.9 months	–	19% vs. 5%	83% vs. 65%	41.6	GENUINE	76% vs. 83%	NCT02301156 [148]
TN CLL	Ibrutinib + fludarabine	2	29	24 (91.3%)	24 (95.8%)	44.4%	93.1%	29	–	–	NCT02514083 [149]
TN CLL	(a) Ibrutinib/(b). chlorambucil + obinutuzumab	3	229	30 (79% vs. 31%)	86% vs. 85%	19% vs. 8%	88% vs. 73%	31.3	iLLUMINATE	58% vs. 35%	NCT02264574 [150]
TN CLL	Ibrutinib + obinutuzumab	2	135	36 (95.7%)	36 (98%)	73.3%	–	36.7	ICLL07 FILO	58%	NCT02666898 [151, 152]
R/R CLL	a. Ibrutinib + BR b. BR	3	578	65.1 vs 14.3	60 (75.7% vs. 61.2%)	40.8% (a)	87.2% vs 66.1%	63.7	HELIOS	77% vs. 74%	NCT01611090 [153, 154]
CLL	Ibrutinib + bendamustine + ofatumumab	2	66	15 (94%)	15 (97%)	6%	92%	–	CLL2-BIO	72%	NCT02689141 [155]
TN CLL	Acalabrutinib	2	99	48 (96)	NR	7	97	53	ACE-CL-001	38%	NCT02029443 [156]
R/R CLL	Acalabrutinib	2	134	45 (62%)	–	4%	94%	41	ACE-CL-001	66%	NCT02029443 [157, 158]
R/R CLL	a. Acalabrutinib b. Ibrutinib	3	533	38.4 vs. 38.4 months	NR	–	77% vs. 81%	40.9	ELEVATE-RR	68.8% vs. 74.9%	NCT02477696 [96]
TN CLL	a. AO b. Acalabrutinib c. Obinutuzumab + chlorambucil	3	535	48 (87% vs. 77.9% vs. 25.1%)	48 (92.9% vs. 87.6% vs. 88.0%)	30.7% vs. 11.2% vs. 13.0%	96.1% vs. 89.9% vs. 82.5%	46.9	ELEVATE-TN	70.2% vs. 49.7% vs. 69.8%	NCT02475681 [159, 160]
R/R CLL	a. acalabrutinib; b. idelalisib plus rituximab or BR	3	310	12 (88% vs. 68%)	12 (94% vs. 91%)	–	81% vs. 75%	16.1	ASCEND	29% vs. 56% (IR) vs. 26% (BR)	NCT02970318 [161]
TN CLL	AO + venetoclax	2	37	NR	NR	38%	100%	27·6	–	–	NCT03580928 [162]
CLL	AO	1b/2	45	TN: 39 (94.4%); R/R: 42 (72.7%)	TN: 39 (100%); R/R: 42 (82%)	TN: 32% R/R: 8%	TN: 95% R/R: 92%	TN: 39% R/R: 42%	–	Naïve: 63% R/R: 77%	NCT02296918 [117]
R/R CLL	Zanubrutinib	2	91	–	12 (95.6)	3.3	84.6	15.1	–	75.8	NCT03206918 [163]
TN CLL	Zanubrutinib	3	109	18 (88.6%)	18 (95.1%)	3.7%	94.5%	18.2	SEQUOIA	48.6%	NCT03336333[164]
TN CLL	Zanubrutinib + obinutuzumab + venetoclax	2	39	–	–	57%	100%	25.8	–	–	NCT03824483[165]
R/R CLL	Pirtobrutinib	1/2	323	–	–	–	62%	6	BRUIN	13%	NCT03740529 [83]
R/R CLL	a. Tirabrutinib b. TI c. TE	1b	53	–	–	7% vs. 7% vs. 10%	83% vs. 93%. vs. 100%	15.5 vs 34 vs. 30.4	–	24.5%	NCT02457598 [166]
R/R MCL	Ibrutinib	2	111	24 (31%)	24 (47%)	23%	67%	26.7	PCYC-1104-CA	–	NCT01236391 [167, 168]
R/R MCL	IR	2	50	36 (87%	36 (94%)	71%	96%	45	–	–	NCT01880567 NCT02427620[169, 170]
Indolent MCL	IR	2	50	36 (93%)	36 (92%)	80%	84%	36	IMCL-2015	–	NCT02682641 [171]
TN MCL	IR + R-HCVAD	2	131	36 (79%)	36 (95%)	87%	98%	42	WINDOW-1	–	NCT02427620 [172]
TN MCL	a. Ibrutinib + BR b. BR	3	523	80.6 vs 52.9	84 (55.0% vs. 56.8%)	65.5% vs. 57.6%	89.7% vs. 88.5%	84.7	SHINE	81.5% vs. 77.3%	﻿NCT01776840 [173]
R/R MCL	IV	2	24	18 (57%)	18 (57%)	62%	71%	15.9	AIM	58%	NCT02471391 [174]
R/R MCL	IR + lenalidomide	2	50	16	22	56%	76%	17·8	PHILEMON	–	NCT02460276 [175]
R/R MCL	a. Ibrutinib b. Temsirolimus	3	280	14∙6 vs. 6.2	30.3 vs. 23.5	23% vs 3%	77% vs. 47%	38.7	RAY	68% vs. 72%	NCT01646021 [176]
R/R MCL	Acalabrutinib	2	124	24 (49%)	24 (72.4%)	43%	81%	26	ACE-LY-004	39%	NCT02213926[177, 178]
R/R MCL	Zanubrutinib	2	86	36 (47.6%)	36 (74.8%)	77.9%	83.7%	35.3	BGB-3111–206	57%	NCT03206970[179, 180]
R/R WM	Ibrutinib	2	63	60 (54%)	60 (87%)	79.4%	90.5%	59	–	30.2%	NCT01614821 [181, 182]
R/R WM	Ibrutinib	3	31	18 (86%)	18 (97%)	71%	90%	18·1	–	65%	NCT02165397 [183]
TN WM	Ibrutinib	2	30	48 (76%)	100%	87%	100%	50	–	–	NCT02604511 [184, 185]
WM	a. IR b. Rituximab	3	150	54 (68% vs. 25%)	54 (86% vs. 84%)	76% v 31%	92% vs. 44%	50	PCYC-1127	60%	NCT02165397 [186, 187]
WM	a. Ibrutinib b. Zanubrutinib	3	201	18 (85% vs. 84%)	18 (93% vs. 97%)	78% vs. 77%	–	19.4	ASPEN	63% vs. 58%	NCT03053440 [97]
WM	Acalabrutinib	2	122	24 (TN: 90%; R/R: 82%)	24 (TN: 92%; R/R: 89%)	MYD88^L265P^: 78%; MYD88^WT^: 57%	93%	27·4	–	53%	NCT02180724 [188]
DLBCL	Ibrutinib	1/2	80	1.64 months	6.41 months	ABC: 16%	ABC: 37%; GCB: 5%	ABC: 10.12; GCB: 17.05	–	–	NCT00849654 NCT01325701 [189]
R/R MZL	Ibrutinib	2	63	15.7 months	33 (72%)	10%	58%	33.1	PCYC-1121	44%	NCT01980628 [190, 191]
R/R MZL	Zanubrutinib	2	68	15 (82.5%)	15 (92.9%)	25.8%	74.2%	15.7	MAGNOLIA	39.7%	NCT03846427 [192]
R/R PCNSL	Tirabrutinib	1/2	44	2.9 months	NR	–	63.6%	9.1	–	–	JapicCTI-173646 [193]
R/R PCNSL	Ibrutinib	2	52	4.8 months	19.2 months	19%	70%	25.7	–	–	NCT02542514 [194]
TN FL	IR	2	80	30 (65%–67%)	30 (97%–100%	40%–50%	75%–85%	29–34	PCYC-1125-CA	64%	NCT01980654 [195]

*N* number enrolled; *PFS* progression-free survival; *OS* overall survival; *CR* complete response; *MRR* major response rate; *ORR* overall response rate; *TN* treatment-naïve; *R/R* relapsed and refractory; *NR* not reached; *IR* ibrutinib plus rituximab; *IV* ibrutinib plus venetoclax; *FCR* fludarabine, cyclophosphamide, and rituximab; *AO* acalabrutinib plus obinutuzumab; *BR* bendamustine plus rituximab; *TI* tirabrutinib plus idelalisib; *TE* tirabrutinib plus entospletinib; *R-HCVAD* rituximab, cyclophosphamide, vincristine, doxorubicin, and dexamethasone

*PFS and OS results are presented as median PFS/OS (months) or *x*-month PFS/OS (%)

However, clinical experience tells us that ibrutinib monotherapy has disadvantages, including a low complete remission rate and undetectable minimal residual disease (uMRD), drug resistance, potential toxicity, and heavy financial burden. Despite the high ORR, CR and MRD eradication are infrequently obtained with single-agent ibrutinib in CLL patients [138, 196]. This indicated that alternative signaling pathways independent of BCR signaling may be activated to support the survival and growth of ibrutinib-treated CLL cells [197]. Moreover, to retain efficacy, it is necessary for continuous ibrutinib treatment in CLL patients until disease progression or the emergence of unacceptable AEs. Sustained adherence to once-daily ibrutinib therapy was associated with extended survival than those missing ibrutinib for eight consecutive days [198]. But continuous ibrutinib administration increased the possibility of long-term toxicities, drug interactions, and the development of acquired resistance. It also reduced patients’ compliance to treatment and elevated financial burdens for patients and society. These limitations urged the exploration of combination therapy, including combining ibrutinib with immunotherapy, CIT, CAR-T cell therapy, and other targeted agents.

In the treatment of CLL patients, the most extensively investigated immunotherapy agents combined with ibrutinib are anti-CD20 agents, including ofatumumab, obinutuzumab, ublituximab, and rituximab. Combination of ibrutinib with ublituximab or ofatumumab was highly tolerable and resulted in a rapid and high response rate (ORR > 83%) in R/R CLL patients [199, 200]. In the multicenter iLLUMINATE phase 3 study, ibrutinib plus obinutuzumab therapy showed significantly prolonged PFS than the standard chlorambucil plus obinutuzumab treatment (30-month PFS: 79% vs. 31%) in previously untreated CLL patients, including high-risk patients with del(17p), del(11q), TP53 mutation and unmutated IGHV [150]. Ibrutinib–obinutuzumab induction therapy followed by an MRD-guided evaluation approach allowed fixed-duration treatment of previously untreated CLL. In the ICLL07 FILO trial, patients who achieved CR with uMRD in bone marrow will be further administered ibrutinib for 6 months. In contrast, those with partial response will receive four cycles of additional CIT (fludarabine, cyclophosphamide, and obinutuzumab) [151]. This strategy led to high PFS and ORR in three years with manageable long-term AEs [151, 152]. Based on the promising results, ibrutinib–obinutuzumab combination has been approved for the management of TN CLL patients in 2019. In the phase 3 E1912 clinical trial, ibrutinib–rituximab combination exhibited superior PFS than the FCR regime (fludarabine, cyclophosphamide, and rituximab) in both IGHV mutated and IGHV unmutated CLL patients [141]. This finding promoted the approval of this combination strategy for previously untreated CLL patients in 2020. However, it was reported that the addition of rituximab provides no additional benefits to ibrutinib monotherapy [138, 201, 202]. Similar results were observed with the programmed cell death protein 1 (PD-1) monoclonal antibody nivolumab [203]. Therefore, these combination regimes should be used with caution owing to the added risk/benefit ratio.

Before the introduction of ibrutinib, CIT regimes like FCR and BR (bendamustine and rituximab) were commonly applied as the standard of care for CLL patients [204]. However, CIT showed limited efficacy in high-risk patients and was related to multiple complications [204]. Ibrutinib was reported to enhance the effectiveness of FCR and BR strategies without additional toxicities in a multicenter phase 1b study [205]. The combination of ibrutinib with FCR (iFCR) is a promising time-limited approach as a frontline treatment for CLL patients without high-risk features. Six cycles of iFCR therapy resulted in 33% CR with uMRD in bone marrow in 2 months, significantly higher than the 20% historical rate with FCR [206]. In a phase 2 clinical trial, patients that received three cycles of CIT followed by nine additional cycles of ibrutinib with three or nine cycles of obinutuzumab achieved 98% of PFS and OS at 3 years. 98% of the patients were negative for MRD at best response [207]. Similarly, in patients suitable for BR treatment, the addition of ibrutinib resulted in improved survival outcomes and deeper response without new safety concerns [153, 154, 208]. In the CLL2-BIO study, sequential treatment of bendamustine debulking combined with ofatumumab and ibrutinib showed an ORR of 92% and acceptable tolerability [155]. These results strongly supported the combination of ibrutinib with different CIT regimes to achieve prolonged survival and deeper remission with a time-limited course in CLL patients.

Ibrutinib can also be combined with targeted therapeutics, such as BCL-2 inhibitor (venetoclax), PI3K inhibitor (umbralisib), and STAT-1 inhibitor (fludarabine), allowing for chemotherapy-free approaches to obtain CR in both previously untreated and R/R CLL patients. Ibrutinib plus venetoclax as first-line treatment induced relatively high rates of uMRD (75% in peripheral blood and 68% in bone marrow) and CR (46%), as well as a 90% reduction in high-risk tumor lysis syndrome [142]. Of the patients achieving uMRD, one-year disease-free survival was similar between patients receiving ibrutinib or placebo treatment, suggesting the induction of treatment-free remissions. Of the patients without confirmed uMRD, continuous therapy with ibrutinib or ibrutinib plus venetoclax achieved a 30-month PFS of over 95% [209]. These results are consistent with that from another two clinical trials, indicating that the addition of venetoclax to ibrutinib induced durable and deep remission in TN CLL patients [143, 144]. Ibrutinib plus venetoclax also demonstrates a high CR rate and encouraging survival outcomes with good tolerability in R/R CLL [145, 146]. Combining therapeutics with different action mechanisms can further optimize the clinical results of ibrutinib plus venetoclax for CLL patients [147, 210, 211]. Two studies are ongoing to determine whether the triplet combination (ibrutinib, venetoclax, and obinutuzumab) is superior to the ibrutinib plus venetoclax regime in managing CLL patients (NCT03701282 and NCT03737981).

Umbralisib [212, 213] and fludarabine [149] have been reported to show synergies with ibrutinib in R/R CLL patients, inducing high rates of CR. These promising results encouraged further exploration of novel combination approaches. A phase 2 clinical trial is ongoing to investigate the efficacy and safety of ibrutinib, fludarabine, and pembrolizumab (anti-PD-1) in managing high-risk or R/R CLL patients (NCT03204188). CAR-T cell therapy has shown excellent responses in some CLL patients. It is observed that ibrutinib treatment increased the expansion of CD19-directed CAR-T cells and reduced the expression of immunosuppressive molecules, including PD-1 on T cells and CD200 on B cells [214]. Ibrutinib exposure also promoted CAR-T cell engraftment and improved tumor clearance and survival outcomes in human xenograft models of CLL [214]. These results indicated that a combination of ibrutinib and CAR-T cells might take advantage of their distinct activities, which is worth investigating in clinical trials.

#### Acalabrutinib

As a representative of the highly selective second-generation BTKi, acalabrutinib showed promising efficacy and safety profiles in different subgroups of CLL patients. 100 mg BID dosing of acalabrutinib was well tolerated and induced a high level of ORR (95.8%) and PFS (24 months: 91.5%) [215]. The ASCEND [161] and ELEVATE-TN [159] phase 3 clinical trials proved that acalabrutinib showed superior efficacy to the traditional treatment approaches (idelalisib plus rituximab, BR, and obinutuzumab + chlorambucil) by providing prolonged PFS in both TN and R/R CLL patients. Long-term observation confirmed the durable efficacy and long-term safety of acalabrutinib for up to 53 months, with an ORR of 97% and a 48-month PFS of 96% [156, 157]. The most common AEs of acalabrutinib were headache, diarrhea, and upper respiratory tract infection [216]. Acalabrutinib demonstrated non-inferior survival outcomes but fewer cardiovascular toxicities than ibrutinib in a randomized phase 3 clinical trial involving 533 patients with previously treated CLL [96]. In patients who were intolerable to ibrutinib and had a persistent disease, acalabrutinib exhibited a high response rate (81%) and favorable safety profiles [217, 218], making acalabrutinib a prior candidate for BTKi therapy in ibrutinib-intolerant CLL patients. In November 2019, the FDA approved acalabrutinib for adults with CLL, irrespective of age and comorbidities. However, it should be noticed that acalabrutinib showed limited efficacy in Richter transformation as monotherapy [219].

Combination studies involving acalabrutinib are also underway. In the ELEVATE-TN clinical trial, the addition of obinutuzumab to acalabrutinib was beneficial with longer PFS in the first-line treatment of CLL [159]. In the phase 1b/2 ACE-CL-003 trial, 19 TN and 26 R/R patients were administered acalabrutinib plus obinutuzumab treatment. After a long-term follow-up for 3.5 years, ORR of 95% in TN and 92% in R/R CLL were observed. At 36 months, the PFS reached 94% and 88% for TN and R/R patients, respectively [117]. These results demonstrated the effective combination of acalabrutinib and obinutuzumab in managing both untreated and R/R CLL. However, a higher frequency of AEs was observed in the acalabrutinib plus obinutuzumab arm, making this combination regime controversial. Acalabrutinib, venetoclax, and obinutuzumab combination therapy could also induce deep and durable remissions with acceptable toxicities as a frontline treatment strategy for CLL patients. After a median follow-up of 27.6 months, 38% of patients achieved a CR with uMRD in the bone marrow [162]. The promising results strongly promoted the evaluation of this triple combination regime in an ongoing phase 3 clinical trial (NCT03836261).

However, Bhat et al. [220] recently reported that long-term administration of acalabrutinib was associated with an eightfold increase in the incidence of ventricular arrhythmias and sudden death events, with a median time to event of 14.9 months. The incidence was higher in patients receiving prior ibrutinib treatment. Therefore, constant surveillance of AEs and early interventions are required for physicians when using acalabrutinib in the clinic. Meanwhile, combined therapy may be recommended, which makes it possible for time-limited therapy with BTKi and allows the treatment to be completed before developing life-threatening AEs.

#### Zanubrutinib

Zanubrutinib demonstrated encouraging activity with a low rate of serious toxicities. No dose-limiting toxicities were observed when CLL patients were orally treated with different doses of zanubrutinib for up to four years in a dose escalation study [221, 222]. Zanubrutinib treatment resulted in a high ORR (84.6%) in 91 Chinese patients with R/R CLL [163]. In TN patients with del(17p) features, zanubrutinib treatment yielded a relatively high ORR (94.5%) in the phase 3 SEQUOIA trial [164], which was non-inferior to that reported in ibrutinib-treated CLL patients [133, 138]. The most common AEs were contusion, airway infection, neutropenia, and diarrhea [164]. A head-to-head phase 3 clinical trial is ongoing to compare the efficacy and safety of zanubrutinib versus ibrutinib in 652 patients with R/R CLL (NCT03734016). An interim analysis indicated that at a median follow-up of 15 months, the ORR of zanubrutinib and ibrutinib were 78.3% and 62.5%, respectively. At 18 months, 20 patients receiving zanubrutinib had disease progression compared to 42 patients receiving ibrutinib. Moreover, zanubrutinib treatment was related to lower rates of atrial fibrillation/flutter (2.5% vs. 10.1%) and AE-caused discontinuation (7.8% vs. 13.0%) [223]. These results suggested that the selective zanubrutinib may be more effective and safer than the standardized ibrutinib in managing CLL patients.

Zanubrutinib can also be combined with immunotherapy or targeted therapy to obtain deep remission with time-limited treatment. In phase 1b study, zanubrutinib plus obinutuzumab treatment induced a deep response by yielding a CR of 28% and 30% in TN and R/R CLL patients, respectively [224], which was significantly higher than that reported by zanubrutinib monotherapy (approximately 3%) [163, 164]. A triplet combination of zanubrutinib, obinutuzumab, and venetoclax was even more powerful as the initial treatment for CLL [165]. This combination reached a high uMRD rate (89%) in both peripheral blood and bone marrow after a 25.8-month follow-up, which may indicate treatment discontinuation [165]. Considering the small sample size (*n* = 39) of this study, additional observation after treatment and clinical trials involving more patients are required to verify the benefits of the three-drug combination strategy in CLL patients.

#### Other inhibitors

Orelabrutinib, pirtobrutinib, and tirabrutinib have shown promising efficacy and safety profiles in early-phase clinical trials in patients with R/R CLL. High ORRs were achieved when patients were treated with single-agent pirtobrutinib (62%) [83], orelabrutinib (91.3%) [225, 226], and tirabrutinib (96%) [227]. In December 2020, orelabrutinib received its first approval in China for CLL treatment with at least one previous treatment. Pirtobrutinib monotherapy showed activity in heavily pretreated CLL patients who underwent BTK^Cys481^ mutant or were resistant/intolerant to BTKi treatment, suggesting its wide therapeutic index and strong efficacy [83]. Although a combination of tirabrutinib and idelalisib or entospletinib, with or without obinutuzumab, showed therapeutic activity and acceptable safety profile, the CR rates remained relatively low (≤ 10%) [166, 228]. Further studies are required to confirm the potential of the novel BTKi and find combination strategies that could induce deep responses in CLL patients. For instance, DTRMWXHS-12 and fenebrutinib are two novel BTKi under clinical investigation to treat R/R CLL patients (NCT04305444, NCT01991184). Multiple phase 3 clinical trials have been launched to compare the efficacy and safety of pirtobrutinib (NCT05023980, NCT04965493, NCT05254743, NCT04666038) and orelabrutinib (NCT04578613) to the conventional CIT regimes, targeted therapy, or ibrutinib. Although the clinical data have not been released yet, the promising preclinical results have indicated their potential activity against CLL, including ibrutinib-resistant CLL [85].

### BTK inhibitors in MCL

MCL is a heterogeneous subtype of B cell non-Hodgkin’s lymphoma (NHL) with distinct clinical courses varying from occasionally indolent to frequently aggressive [229]. Although the intensive chemotherapy or CIT followed by autologous hematopoietic cell transplant showed a high response rate, most patients would experience relapse and chemoresistance, leading to eventual death [229]. Developing more effective and less toxic treatment is necessary, especially for older patients with R/R MCL. BTK is commonly overexpressed in MCL cells. Inhibition of BTK with ibrutinib induced apoptosis and reduced adhesion and migration of MCL cells via BCR or chemokine signaling pathways [52, 230]. Constitutive activation of LYN, BLNK, SYK, PKCβ, and NF-κB was also observed in MCL and was correlated with the survival [231–234]. These results provided the theoretical basis for targeting BTK as a promising therapeutic modality for MCL. The development of BTKi has revolutionized MCL treatment, which showed high activity and tolerability in both previously untreated and R/R MCL patients as monotherapy or combined therapy. At present, four BTKi (ibrutinib, acalabrutinib, zanubrutinib, and orelabrutinib) have received their approval for the treatment of R/R MCL patients after at least one prior therapy. Current efforts are focused on BTKi as a component of combined therapy to induce deep response as both salvage and frontline therapy.

#### Ibrutinib

Ibrutinib represents a remarkable advance in treating R/R MCL, with an ORR of over 68% and an estimated median PFS of 13.9 months [167, 235]. Long-term observations indicated that ibrutinib induced durable response and favorable safety profiles at a median follow-up of 26.7 months [168]. Moreover, ibrutinib showed superior ORR (77% vs. 47%), CR (23% vs. 3%), and PFS (14∙6 vs. 6.2 months) than the mTOR inhibitor temsirolimus in the management of R/R MCL. These promising results have promoted the approval of ibrutinib for the treatment of R/R MCL in 2013. However, ibrutinib monotherapy was ineffective in inducing CR. Most MCL patients became ibrutinib-resistant in 10–14 months and developed a poor prognosis after treatment failure [236]. Combining ibrutinib with immunotherapy, CIT, or targeted therapy may improve the outcomes in both TN and previously treated MCL patients. Among different combination regimes, ibrutinib plus anti-CD20 rituximab or BCL-2 inhibitor venetoclax has garnered considerable interest.

Clinical data from phase 2 studies indicated that ibrutinib plus rituximab-induced durable and high response and prolonged survival in patients with TN [170], R/R [169, 237], and indolent [171] MCL. The CR rate (44–80%) and PFS (3-year survival 87%) were significantly elevated than that reported with single-agent ibrutinib, although the head-to-head comparison is not available [167, 235]. This combination regime also yielded 87% cases of uMRD in the peripheral blood of indolent MCL patients, contributing to the discontinuation of ibrutinib [171]. It should be noted that the efficacy of this combination may be reduced in the context of TP53-mutation and Ki-67 high expression [171, 237]. Ibrutinib–rituximab induction followed by shortened R-HCVAD CIT regime induced an extremely high rate of overall response (98%) as frontline treatment in young MCL patients, with reduced chemotherapy-related AEs [172]. The addition of bendamustine [238] or lenalidomide [175] into the ibrutinib–rituximab regime showed promising activity and tolerability. In the phase 3 SHINE study, ibrutinib plus the BR regime resulted in a drastic prolongation of 2.3 years in the median PFS in TN older MCL patients (≥ 65 years old) after a median follow-up of 7 years compared with patients treated with only BR [173]. These results strongly supported the addition of ibrutinib to the standard first-line BR treatment regime for an increased opportunity of durable disease control to inhibit or delay relapse in older MCL patients who are unsuitable for autologous stem cell transplantation.

Ibrutinib plus venetoclax treatment induced high levels of CR (42–62%) and MRD clearance (67%) with no new safety concerns even in MCL patients with predictors of poor outcomes (with TP53 mutation or high-risk prognostic score) [174, 239]. These promising results supported the initiation of the phase 3 SYMPATICO study to compare the efficacy and safety of ibrutinib + venetoclax versus ibrutinib monotherapy in TN MCL patients (NCT03112174). The addition of obinutuzumab to ibrutinib–venetoclax was also well tolerated and resulted in durable CR in previously untreated and R/R MCL patients with high-risk genetics [240]. In addition, ibrutinib can be combined with other agents, including PI3K inhibitor umbralisib [212] and buparlisib [241], cyclin-dependent kinase 4/6 (CDK4/6) inhibitor palbociclib [242], and proteasome enzyme inhibitors carfilzomib [243], which showed preliminary activities and good tolerability in R/R MCL patients in early-phase studies. Whether these combinations are superior to ibrutinib alone remains to be confirmed.

#### Acalabrutinib

Acalabrutinib showed encouraging benefits in the multicenter phase 2 ACE-LY-004 study, which promoted its accelerated approval by the US FDA in 2017 to treat adult MCL patients with at least one previous therapy. Acalabrutinib monotherapy provided durable and clinically meaningful responses (ORR 81%, CR 40%) and survival benefits (12-month PFS: 67%) in R/R MCL patients [178]. These findings demonstrated its superior efficacy to other licensed agents, including ibrutinib, lenalidomide, bortezomib, and temsirolimus (ORR 22–68%, CR 2–21%) [244]. Extended follow-up for 26 months verified the continued efficacy and tolerability of acalabrutinib in R/R MCL, including those with high-risk features of poor prognosis (Ki-67 index ≥ 50%) [177]. Acalabrutinib also achieved 28% of MRD negativity, a strong indicator of clinical outcomes in MCL [177]. Despite the promising results, ACE-LY-004 is a single-arm study which may have overemphasized the outperformance of acalabrutinib without the head-to-head comparison between acalabrutinib and other agents including ibrutinib. The clinical trial of ibrutinib versus acalabrutinib in CLL indicated their relative safety. Still, the findings cannot be easily extrapolated to MCL patients who received a higher dose of ibrutinib than CLL patients (560 mg vs. 420 mg) [96]. Moreover, twice-daily dosing may impair patient compliance and impact the efficacy of acalabrutinib in reality. The results of an ongoing phase 3 clinical trial (NCT02972840) may help evaluate the values of acalabrutinib, which compares bendamustine–rituximab combination with or without acalabrutinib in TN MCL patients. Combinations of acalabrutinib with other therapies are also under intensive investigation in MCL patients, including chemotherapy (NCT04566887), immunotherapy (NCT05004064, NCT04765111, and NCT05214183), targeted therapy (NCT04783415), and CAR-T cell therapy (NCT04484012).

#### Zanubrutinib

Zanubrutinib is efficacious in R/R MCL patients, which could induce durable and deep remission as a single agent. Phase 1 clinical trials conducted in China (BGB-3111–1002) and other countries (BGB-3111-AU-003) have proved the safety profiles of zanubrutinib in the treatment of MCL without dose-limiting toxicities at doses up to 320 mg daily [245]. Later in the phase 2 BGB-3111–206 study, zanubrutinib demonstrated an ORR of 84% in 86 patients, including 68.6% of patients who achieved CR [180]. Although 57% of patients developed grade ≥ 3 AEs, only 9.3% discontinued zanubrutinib treatment, suggesting a favorable safety profile for zanubrutinib [180]. Long-term follow-up of this study for a median of 35.3 months confirmed the deep and durable response, extended PFS (median 33.0 months), and good tolerability of zanubrutinib [179]. Based on the promising results, zanubrutinib received accelerated approval in the US in late 2019 for treating R/R MCL patients with at least one prior therapy. A phase 3 study (BRUIN MCL-321) is ongoing to compare zanubrutinib with other BTKi in 500 previously treated MCL patients, including ibrutinib, acalabrutinib, and pirtobrutinib (NCT04662255). The results would greatly assist the overall assessment of zanubrutinib in MCL. In addition, there are plenty of ongoing clinical trials investigating zanubrutinib as part of two- or three-drug combinations in the treatment of MCL (NCT04002297, NCT04624958, NCT03824483, etc.)

#### Other inhibitors

In the ICP-CL-00102 clinical trial, orelabrutinib exhibited excellent safety profiles and pharmacokinetic/pharmacodynamic properties in R/R MCL patients [246]. When dosed with 150 mg once daily, orelabrutinib demonstrated strong efficacy of R/R MCL with an ORR of 82.5% and a CR of 24.7% [246]. After extended treatment for 15 months, the response remains to be strong (CR 27.4%) with only a mild increase in the AEs [247]. The high potency, good tolerability, and convenience of once-daily dosing make orelabrutinib an attractive therapeutic option. Based on the promising results, orelabrutinib was conditionally approved for the treatment of R/R MCL in China in December 2020. The full approval relies on the confirming efficacy from the ongoing clinical trials (NCT05051891, NCT05076097, and NCT05097443). Current data from phase 1/2 trials also revealed the efficacy and safety of the third-generation non-covalent BTKi pirtobrutinib in R/R MCL patients who have received covalent BTKi [83]. These results suggested that pirtobrutinib can be used as an alternative strategy in patients resistant to previous BTK inhibition therapy. A phase 3 clinical trial is also underway to compare the efficacy of pirtobrutinib to the approved BTKi in MCL patients (NCT04662255). Other promising BTKi that are under evaluation in early-phase clinical trials against MCL include DTRMWXHS-12 (NCT03836768) and nemtabrutinib (NCT03162536). Their success will eventually rely on the results from the clinical studies assessing their efficacy and safety.

### BTK inhibitors in WM

WM is a rare and indolent B cell lymphoma characterized by the infiltration of bone marrow and lymphatic tissues with lymphoplasmacytic cells, which could generate monoclonal immunoglobulin M (IgM) in the serum [248]. Whole-genome sequencing indicated that about 93–97% of WM patients have a somatic mutation in MYD88, namely MYD88^L265P^ [181]. This disorder triggers tumor growth by activating NF-κB signaling via BTK. Constitutive activation of BTK was observed in WM secondary to MYD88 mutations [249]. MYD88 mutation can also transactivate hematopoietic cell kinase (HCK), a pro-survival factor and highly relevant target for ibrutinib [250]. Additionally, CXCR4^WHIM^ mutations were almost exclusively observed in WM patients with the MYD88^L265P^ variant, which contributed to ibrutinib resistance [181]. These results supported the application of BTKi in the management of WM and addressed the importance of routine detection of gene mutations before treatment initiation.

#### Ibrutinib

In R/R MCL patients, ibrutinib induced a fast response with a median time to the first response of 4 weeks [181]. The ORR and major response rate (MRR, refers to partial or very good partial response (VGPR)) were highest among patients with MYD88^L265P^CXCR4^WT^ (100% and 91.7%, respectively), followed by MYD88^L265P^CXCR4^WHIM^ patients (85.7% and 61.9%), and lowest in patients with unmutated MYD88 (60% and 0%) [181]. Based on these results, the US FDA and European Medicines Agency (EMA) approved ibrutinib for symptomatic WM, which significantly altered the management and outcome landscape of this malignancy. Five-year follow-up revealed that the PFS decreased from 70% for MYD88^L265P^CXCR4^WT^ patients to 38% for MYD88^L265P^CXCR4^WHIM^ patients [182]. Ibrutinib also showed high potency in TN WM patients carrying MYD88 mutation, with an ORR and MRR of 100% and 83%, respectively [184]. In this study, patients with WT CXCR4 showed more rapid (1.8 vs. 7.3 months) and higher MRR (94% vs. 71%) than those with mutated CXCR4. The 4-year PFS rate achieved 76% [185], suggesting the induction of an effective and durable response by ibrutinib. Bing–Neel syndrome (BNS) is an uncommon presentation of WM when malignant cells enter the central nervous system and cause neurological disorders [251]. In s retrospective study, ibrutinib showed rapid and long-term responses in BNS patients both symptomatically and radiologically, with 2-year event-free survival reaching 80% [252]. Therefore, single-agent ibrutinib is highly effective and tolerable, which could induce durable responses in TN, R/R WM (including heavily pretreated and rituximab-refractory cases [183]), and BNS patients. The effect of ibrutinib is influenced by the mutation status of MYD88 and CXCR4. Patients with MYD88^WT^ are not suitable for ibrutinib monotherapy since no major response was observed.

However, the dependence on the mutation status of MYD88 and CXCR4 has limited the application of ibrutinib. In addition, treatment discontinuation for over seven days resulted in fourfold increase in progression [253], highlighting the importance of consistent ibrutinib therapy in WM. Researchers have focused on using the second-generation BTKi or exploring effective combination regimes to tackle these dilemmas. In the phase 3 iNNOVATE clinical trial, 150 TN and R/R WM patients were randomized (1:1) to receive rituximab-placebo or rituximab–ibrutinib treatment. The addition of rituximab to ibrutinib led to higher MRR (72% vs. 32%) and prolonged PFS (82% vs. 28% at 30 months) in both TN and R/R patients, regardless of the MYD88 and CXCR4 genotypes [186]. Moreover, rituximab–ibrutinib treatment rapidly reduced the IgM levels in the serum and prevented rituximab-induced IgM flare [186]. The clinical benefits of ibrutinib–rituximab were sustained after a median follow-up of 50 months [187]. In 2018, ibrutinib–rituximab combination received its approval for WM patients. Despite promising, it should be noticed that the frequency of grade ≥ 3 hypertension, atrial fibrillation, and pneumonia was increased in patients receiving ibrutinib–rituximab treatment. Moreover, no patients in the study received ibrutinib monotherapy, making it difficult to assess the additional benefits of rituximab. As mentioned earlier, CXCR4 mutation induced drug resistance and compromised the sensitivity of WM patients to ibrutinib treatment. Thus, blocking CXCR4 signaling with specific inhibitors may rescue the decreased benefits caused by CXCR4 mutation. In phase 1 study, ibrutinib plus CXCR4 antagonist ulocuplumab yielded an MRR and VGPR of 100% and 33%, respectively [254].

#### Zanubrutinib

Zanubrutinib monotherapy has displayed deep and durable efficacy and long-term tolerability in TN and R/R WM patients in early clinical trials [255, 256]. The estimated 3-year PFS reached up to 80.5% [255]. In the phase 3 ASPEN study, zanubrutinib showed superior potency, lower treatment-related AEs, especially cardiovascular toxicity, and decreased treatment discontinuation than ibrutinib in WM patients [97]. Of note, zanubrutinib showed high-quality responses (MRR 50%; VGPR: 27%) and survival outcomes (18 months PFS 68%) in WM patients with MYD88^WT^ [257], unlike the observed 0% of MRR in ibrutinib-treated patients. Nevertheless, the MRR in MYD88^WT^ patients was still lower than that of the MYD88^L265P^ population [256]. Therefore, zanubrutinib is highly responsive across all subtypes of WM patients, which promoted its accelerated approval for WM treatment in 2021.

#### Other inhibitors

Some other promising BTKi under clinical development for the treatment of WM include acalabrutinib and tirabrutinib. In a single-arm multicenter phase 2 study, acalabrutinib treatment exhibited an ORR of 93% and 86% in TN and R/R WM patients with a manageable safety profile [188]. Further studies are necessary to investigate the influence of MYD88 and CXCR4 mutations, long-term efficacy and safety, its benefits over the first-in-class ibrutinib, and possible combination strategies. In another phase 2 study, tirabrutinib monotherapy showed encouraging activity (MRR, 88.9%) and acceptable safety in both TN and R/R WM patients [258]. After follow-up for 24.8 months, the MRR increased to 93%, with a 24-month PFS of 92.6% [259]. Therefore, tirabrutinib is a fascinating alternative BTKi and deserves further exploration for WM treatment.

### MZL

MZL is a heterogeneous B cell malignancy derived from memory B cells in the marginal zones. It is closely related to antigen-mediated BCR activation in autoimmunity and chronic infection [260], suggesting BTK as a potential therapeutic target. MZL can be divided into nodal, extra-nodal, and splenic subtypes [261]. In the phase 2 PCYC-1121 study, single-agent ibrutinib was highly active with a favorable benefit–risk profile in all subtypes of R/R MZL who have previously received rituximab-based treatment. After a median follow-up for 19.4 months, ibrutinib treatment induced an ORR of 48% and a median PFS of 14.2 months [190]. The ORR elevated up to 58% after 33.1 months of continuous ibrutinib treatment, suggesting long-term efficacy [191]. Based on these findings, ibrutinib obtained its accelerated approval in 2017 for R/R MZL patients with at least one prior treatment based on anti-CD20 therapy. A phase 3 study (SELENE) is currently evaluating a combination of ibrutinib with BR or R-CHOP regimes in the management of indolent NHL, including MZL, with pending results (NCT01974440).

Zanubrutinib was also approved by FDA for the treatment of R/R MZL patients in 2021, which was based on two multicenter clinical trials: BGB-3111-AU-003 and BGB-3111–214 [262]. After a median follow-up of 15.9 months, zanubrutinib monotherapy induced an ORR of 74.2%, a CR of 25.8%, and a PFS of 82.5% at 15 months [192], higher than that reported by single-agent ibrutinib at the same period [190]. In addition, zanubrutinib is better tolerated with a lower rate of serious or grade ≥ 3 AEs (39.7% vs. 44%). Therefore, zanubrutinib may be better for R/R MZL patients with higher potency and tolerability than ibrutinib.

### ABC-DLBCL

DLBCL is the most common type of aggressive NHL. It has profound molecular heterogeneity defined by different gene expression patterns and various mechanisms of oncogenic activation [263]. DLBCL can be divided into GCB and ABC subtypes. Non-GCB-DLBCL is a rough but imperfect approximation of ABC-DLBCL [264]. ABC-DLBCL patients have a poorer prognosis than GCB-DLBCL following traditional chemotherapy [263]. In ABC-DLBCL, constitutive activation of BCR and NF-κB signaling was associated with lymphomagenesis and cancer cell survival [265, 266]. About half of ABC-DLBCL possess mutations in CARD11 or other components of the NF-кB signaling pathway, including the MYD88^L265^ variant [267, 268]. Additionally, 1/5 of ABC-DLBCL patients harbor an activating mutation in CD79a/b. Knockdown of BTK, CD79a*,* and IgM selectively killed ABC-DLBCL cell lines. These signaling differences accounted for the distinct responses of ABC-DLBCL and GCB-DLBCL patients to ibrutinib monotherapy (37% vs. 5% of CR or partial response (PR)) in the phase 1/2 study [189].

The efficacy of BTKi is modest in ABC-DLBCL, not comparable to currently available therapies with a cure rate of ~ 40% [269]. In the PHOENIX phase 3 trial, the addition of ibrutinib to R-CHOP chemotherapy showed no evidence of improved responses and survival but more toxicities in untreated non-GCB-DLBCL patients [270], consistent with that discovered in another phase 2 study [271]. One reason for the disappointing results may be the less accurate classifier, the immunohistochemistry method. Wilson et al. analyzed the biopsy samples of younger patients (≤ 60 years) from the PHOENIX study and classified them into different genetic subtypes: MCD, BN2, and N1. They found that the 3-year event-free survival of patients in the MCD and N1 subgroups was significantly higher when treated with ibrutinib plus R-CHOP (100%) than R-CHOP alone (42.9%-50%) [272]. These findings supported the application of ibrutinib combined R-CHOP therapy for younger patients with MCD and N1 characteristics. Ibrutinib is also under investigation for combining other chemotherapy [273, 274] or immunotherapy [203, 275, 276] regimes, with the ORR ranging from 38 to 90% in early clinical trials. As for the second-generation BTKi, zanubrutinib monotherapy showed an ORR of 29.3% and a median PFS of only 2.8 months in the phase 2 BGB-3111-207 study [277]. The ORR of acalabrutinib monotherapy was similar (33%) in R/R ABC-DLBCL patients [278]. Most patients with the ABC phenotype had a short survival outcome. Thus, a more precise understanding of the molecular mechanisms under the resistance and short duration of response is necessary to identify patients who would benefit from this treatment. Further studies may concentrate on exploring mechanism-based combination strategies and biomarker-guided patient selection.

PCNSL is an aggressive type of DLBCL, which mainly affects the brain, spinal cords, meninges, cerebrospinal fluids, and eyes. The vast majority of PCNSL belongs to the ABC-DLBCL phenotype [279]. Among different BTKi, ibrutinib and tirabrutinib are most extensively investigated as frontline or salvage treatments for PCNSL, either as a single agent or a part of combination methods. In 2020, oral tirabrutinib was licensed to manage R/R PCNSL in Japan. Tirabrutinib induced an ORR of 63.6% and a median PFS of 2.9 months with favorable safety profiles in 44 Japanese patients [193]. More convincing results are needed to promote the broad application of tirabrutinib for R/R PCNSL patients in more countries. Ibrutinib also showed desirable efficacy in the management of PCNSL. In phase 1 clinical study, ibrutinib treatment induced a high ORR (77%) in R/R PCNSL patients [280], which is much higher than that reported in DLBCL outside the CNS, indicating divergent molecular pathogenesis [280]. Later in a multicenter phase 2 study, 70% of PCNSL patients receiving ibrutinib treatment achieved disease control after 2 months of therapy [194]. The median PFS and OS were 4.8 months and 19.2 months, respectively, higher than that reported by tirabrutinib [194]. The addition of ibrutinib to anthracycline-based chemotherapy significantly improved the ratio of CR (86%) [281]. But we should notice that this combination was associated with undesirable toxicity, including the high frequency of aspergillosis infection. A combination of ibrutinib with CIT (rituximab plus methotrexate [282, 283] or lenalidomide [284]) induced sustained antitumor response in R/R PCNSL patients both in clinical trials and in the real world, including the heavily pretreated cases. Therefore, the application of BTKi is a promising strategy in PCNSL patients, either as monotherapy or in combination.

### Follicular lymphoma (FL)

FL is an indolent lymphoproliferative disorder of follicular center B cells, which is generally incurable with standard CIT [285]. Increased BCR activation is observed in FL cells through an antigen-dependent or independent manner, promoting the application of BTKi in the treatment of FL. In phase 2 study of 80 TN FL patients, the ibrutinib–rituximab combination demonstrated favorable activity and tolerability as a frontline treatment, with an ORR of 75–85%, a CR of 40–50%, and a 30-month PFS of 67% [195]. In R/R patients, however, ibrutinib showed limited efficacy over rituximab-based therapy either as a single agent [61, 286] or in combination [241, 275, 287]. But the remained rationale supported further exploration of more advanced BTKi. Zanubrutinib, for example, was observed to be more tolerated and induced a slightly higher response rate than ibrutinib in R/R FL patients [288]. The addition of proper therapies to zanubrutinib, e.g., Obinutuzumab [224], may be beneficial and improve the outcomes of R/R FL. Another second-generation BTKi spebrutinib is now under a multicenter phase 1 clinical trial to treat FL in combination with rituximab (NCT02031419).

### Multiple myeloma (MM)

MM is a malignancy of terminally differentiated plasma cells characterized by heterogeneous cytogenetic abnormalities [289]. BTK is overexpressed in > 85% of tumor cells and can increase the stemness properties of cancer cells from MM patients, including self-renewal, clonogenicity, and drug resistance [290]. In early-phase clinical trials in R/R MM patients, ibrutinib produced notable responses (ORR ranging from 28 to 71%) and acceptable safety profiles when combined with the classic therapeutics, including dexamethasone [291], bortezomib [292, 293], and carfilzomib [293, 294]. The recommended dose of ibrutinib for MM is 840 mg once daily, higher than that used in other hematological malignancies (420 mg or 560 mg). The added dosage may increase the risk of treatment-related toxicities. Safety evaluation should be emphasized in further studies, including long-term safety monitoring.

## BTK inhibitors in inflammatory diseases

As aforementioned, apart from B cells, BTK is highly expressed in many other immune cells, including T cells, monocytes–macrophage, neutrophils, and mast cells. Overactivation of BTK in these cells activates multiple signaling pathways to promote the pathogenesis of inflammatory diseases, including autoimmune diseases, infections, and chronic inflammatory diseases. Currently, both preclinical and clinical data have demonstrated that BTKi showed rapid anti-inflammatory function, neutralized pathogenic autoantibodies, and inhibited the generation of novel self-active antibodies (Table 3). Thus, BTK is an appealing therapeutic target for inflammatory disorders. The last decade has witnessed the triumph of the first-in-class BTKi in managing hematological malignancies. However, the off-target effects of ibrutinib lead to unfavorable safety profiles. Most patients with inflammatory disease manifest mild-to-moderate symptoms and require more selective inhibitors with fewer side effects. Thus, although not approved, current studies of BTKi in inflammatory diseases focused on the highly specific next-generation inhibitors.

**Table 3:** Application of BTK inhibitors in inflammatory diseases

Disease	Preclinical studies	Clinical trials
SLE	1. Reduced accumulation of T cells, B cells, macrophages; 2. Reduced production of autoantibodies and inflammatory cytokines; 3. Improved cognitive function in brain disease of lupus [295–297]; 4. Reduced proteinuria, improved glomerular pathology scores and survival in lupus nephritis [295, 296]	Fenebrutinib monotherapy (Phase 2): 1. Reduced anti-dsDNA autoantibodies, total IgG, and IgM levels; 2. Acceptable safety profile; 3. Limited benefits in SRI- 4 response rates: 52% for fenebrutinib vs. 44% for placebo [21]
RA	1. Inhibit B cell proliferation and the production of autoantibodies [22]; 2. Inhibit FcγRIII-dependent production of cytokines (e.g., TNF-α, IL-1β, and IL-6) from macrophages [22]; 3. Reduced paw swelling, histological improvement without body weight loss in CIA [88, 298]	Spebrutinib monotherapy (Phase 2a): Over 20% improvement in ACR response criteria in active RA patients with good tolerability [101] Fenebrutinib monotherapy (phase 2): Showed equivalent efficacy (36%) with adalimumab when fenebrutinib was administered at a dose of 200 mg twice daily [299]
MS	Ex vivo BTK inhibition helped abolish the aberrant activation and expression of costimulatory molecules on B cells from untreated MS patients [300]	Tolebrutinib (phase 2b): reduced the number of new gadolinium-enhancing lesions in a dose-dependent manner in RMS patients [73] Evobrutinib (phase 2): 1. Reduced the number of enhancing MRI lesions in RMS patients at 75 mg once daily; 2. Showed no benefits on the annualized relapse rates or disability deterioration; 3. Elevated liver aminotransferase levels [23]
Pemphigus	1. Reduced anti-desmoglein IgG antibody titers; 2. Rapid reduction in lesions and Pemphigus Disease Activity Index score in the first 2 weeks; 3. Complete or sustained disease control by 20 weeks in canine models [107, 301]	Rilzabrutinib (phase 2 BELIEVE study): .1. CR: 15% by week 12 and 22% by week 24; 2. A reduction in mean prednisone-equivalent corticosteroid: 20.0 to 11.8 mg/d for naive patients; 10.3 to 7.8 mg/d for relapsing patients; 3. Mostly mild treatment-related AEs [302] Tirabrutinib (phase 2): 1. CR: 18.8% by week 24 and 50.0% by week 52; 2. A reduction in mean prednisone-equivalent corticosteroid: 17.03 to 7.65 mg/day [303]
CSU	–	Fenebrutinib (phase 2): dose-dependent improvements in UAS7 in week 8 in antihistamine-refractory patients [304]
ITP	–	Rilzabrutinib (phase 1/2): 1. An overall response in 40% patients after a median of 167.5 days; 2. Only low-level toxicities [91]
Severe COVID-19	1. Ibrutinib and zanubrutinib may interfere with viral entry and replication [305]; 2. Prevent thromboinflammation via direct or indirect interactions with platelets [306]	Acalabrutinib: 1. Normalized inflammation C-reactive protein, IL-6, and lymphopenia; 2. Improved oxygenation within 1–3 days; 3. No discernable toxicity [307]

SRI-4: a composite endpoint used in SLE clinical trials; CIA: collagen-induced arthritis; ACR: is scored as a percentage of improvement; UAS7: urticaria activity score over 7 days

### BTK inhibitors in autoimmune diseases

#### SLE

SLE is characterized by loss of immune tolerance and overproduction of autoantibodies against self-antigens, followed by the formation of immune complexes that contribute to inflammation and tissue damage in multiple organs [308]. An elevated expression and activation of BTK were observed in SLE patients, along with an activation of BCR and FcR signalings in B cells and myeloid cells [309]. These responses promoted cell activation and differentiation, culminating in enhanced production of autoreactive antibodies and inflammatory cytokines, including anti-DNA antibodies [296, 310]. Murine studies indicated that inhibition of BTK significantly limited the infiltration and accumulation of T cells, B cells, and macrophages [295–297]. Correspondingly, the production of autoantibodies and cytokines was decreased in a dose-dependent manner. BTK inhibition successfully reversed established mouse models of SLE disease in the brain [297] and kidney [295, 296]. In a multicenter phase 2 study, 260 patients with moderately to severely active SLE were randomized to receive a placebo or different doses of the non-covalent BTKi fenebrutinib [21]. By week 48, patients in the fenebrutinib group showed dropped levels of anti-dsDNA autoantibodies, total IgG, and IgM compared to the control group. Despite the potent pathway inhibition, fenebrutinib treatment showed limited enhancement in the SRI-4 response rates (52% vs. 44%). Current clinical trials are ongoing to evaluate the efficacy and safety of other selective BTKi in SLE patients alone or in combination, including zanubrutinib (NCT04643470), orelabrutinib (NCT04305197), branebrutinib (NCT04186871), elsubrutinib (NCT03978520, NCT03978520), and AC0058TA (NCT03878303), aiming to search for a more potent therapeutic candidate.

#### RA

RA is a multifactorial autoinflammatory disease featured by synovial membrane hyperplasia, cartilage destruction, and bone erosion. A lower incidence of CIA was observed in mice lacking the BTK gene, indicating the critical role of BTK in the development of RA [311]. Inhibition of BTK with small molecule inhibitors resulted in reduced paw swelling without a bodyweight loss [88, 298]. Mechanism studies revealed that BTK regulated inflammatory arthritis via modulating B cells and myeloid cells in CIA models. Specific BTK inhibition suppressed BCR-dependent proliferation of B cells and diminished autoantibody titers. It also prevented inflammatory cytokine production in macrophages via FcγRIII signaling, including TNF-α, IL-1β, and IL-6 [22]. These results suggested BTK as a promising target for RA treatment. In a phase 2a study, oral treatment with spebrutinib significantly decreased serum levels of CXCL13 and MIP-1β in 47 patients with active RA, which is closely related to more than 20% improvement in ACR response criteria over placebo-treated patients [101]. Another phase 2 study compared the safety and efficacy of fenebrutinib to adalimumab in active RA patients who were inadequately responsive to methotrexate treatment [299]. Although fenebrutinib and adalimumab showed overlapping and distinct impacts on B cell and myeloid cell biomarkers, they showed comparable efficacy (36%) when fenebrutinib was administered at a dose of 200 mg twice daily. At present, another five BTKi are under phase 2 clinical trials for the management of active RA patients, including acalabrutinib (NCT02387762), evobrutinib (NCT03233230, elsubrutinib (NCT03682705), BMS-986142 (NCT02638948), Branebrutinib (NCT04186871). The former three studies have been completed with the results about to be reported.

#### MS

MS is a chronic autoimmune inflammatory demyelinating disorder that causes neurological morbidity and gradual disability [89]. Small molecular BTKi may be more advantageous than monoclonal antibody therapy with fewer toxicities, such as antibody response and allergic reactions. Moreover, some BTKi can penetrate the blood–brain barrier and inhibit the activation of autoreactive B cells and microglial cells in the CNS, which are the primary triggers of inflammation in MS [73, 300]. Recently, oral administration of the brain-penetrant tolebrutinib has shown promising results in relapsing MS (RMS) patients in a phase 2b study [73]. After a 12-week tolebrutinib treatment, there was a dose-dependent reduction in new gadolinium-enhancing lesions. The drug was highly safe without any toxicity-related treatment discontinuation [73]. These results supported the rationale to initiate a long-term follow-up study (NCT03996291) and multiple phase 3 clinical trials (NCT04411641, NCT04458051, NCT04410991, and NCT04410978) of tolebrutinib in relapsing or progressive MS patients. In another phase 2 study, the selective irreversible BTKi evobrutinib reduced the number of enhancing MRI lesions in RMS patients when dosed at 75 mg once daily, rather than 75 mg twice daily or 25 mg once daily. Notwithstanding, evobrutinib treatment had no benefits on the annualized relapse rates or disability deterioration [23]. Extended and large studies are necessary to assess its efficacy and safety profiles in MS. Two phase 3 investigations have now been launched involving 930 RMS patients (NCT04338061 and NCT04338022). Another two promising BTKi, fenebrutinib (NCT04586023, NCT04586010, and NCT04544449) and remibrutinib (NCT05147220 and NCT05147220), have reached phase 3 clinical trials in MS, with results expected before 2024. Orelabrutinib is still in phase 2 clinical trial (NCT04711148) without clinical data at present.

#### Pemphigus

Pemphigus is a chronic autoimmune disease of blistering on the skin and mucosal membranes caused by anti-desmoglein autoantibodies [312]. Preclinical studies indicated that BTKi could induce rapid anti-inflammatory effects in the first two weeks and promote complete or sustained disease control by 20 weeks in canine models of pemphigus [107, 301]. A decrease in the titers of anti-desmoglein IgG antibodies was also detected [301]. These promising results prompted the initiation of the phase 2 BELIEVE study in 27 pemphigus vulgaris patients. In this study, the reversible rilzabrutinib induced rapid clinical activity showing a CR ratio of 15% by week 12 and a reduction in mean prednisone-equivalent corticosteroid [302]. Similarly, the selective tirabrutinib showed high efficacy in relapsed pemphigus patients in Japan, with a CR rate of 18.8% by week 24 and 50.0% by week 52 [303]. Therefore, selective BTKi allowed deep remission over time and reduced oral corticosteroid exposure without any serious safety concerns in pemphigus patients.

#### Other autoimmune diseases

Overactivation of BTK is also of great importance in other autoimmune diseases, including Sjögren’s syndrome [313], neuromyelitis optica spectrum disorders (NMOSD) [314], ITP [315], etc. For instance, in ITP, BTKi enhanced platelet numbers by ameliorating Fcγ receptor-mediated platelet destruction by macrophages and decreasing autoantibody generation [91]. In phase 1/2 clinical trial of 60 previously treated ITP patients, oral rilzabrutinib treatment induced an overall response in 40% of patients after 167.5 days. Only low-level toxicities were observed, without treatment-associated bleeding or thrombotic affairs of grade 2 or higher [91]. These promising results supported the advancement into a phase 3 study to evaluate rilzabrutinib in adults and adolescent patients with persistent or chronic ITP (NCT04562766). In CSU, BTK played a vital role in FcγRI-mediated mast cell activation and promoted the production of autoantibodies in B cells. Inhibition of BTK with fenebrutinib diminished disease activity in CSU patients in a dose-dependent manner at week 8, who were refractory to antihistamine therapy [304]. Selective BTKi tolebrutinib and rilzabrutinib are also under phase 2–3 clinical trials to assess their efficacy and safety in Myasthenia gravis (NCT05132569), IgG4-related disease (NCT04520451), warm autoimmune hemolytic anemia (NCT04520451), and primary membranous nephropathy (NCT05136456).

### BTK inhibitors in infections

Although some studies have investigated the role of BTK in microbial infections, a lot of aspects still remain elusive. Some of the results are controversial and opposite when it comes to different types of microorganisms. Here, we discussed the involvement of BTK signaling and the therapeutic potential of BTKi in viral, bacterial, and fungal infections.

#### Viral infection

BTK plays a crucial role in regulating the innate immune system against viral infection. TLRs expressed in/on macrophages can sense viral RNA and promote the generation of cytokines, chemokines, and phagocytosis via BTK-dependent NF-κB signaling [316]. In influenza A virus infection, BTK overexpression was associated with severe inflammation during the acute lung injury stage [317]. Intranasal administration of ibrutinib significantly attenuated lung inflammation, reduced weight loss, and prolonged survival in mice infected with a lethal dose of influenza virus. In addition, silencing or inhibiting BTK enhanced viral clearance by promoting the death of infected cells [318]. These results highlighted that BTK could be a potential drug target for viral infections. However, we should notice that the impact of BTKi on innate and humoral immunity may increase the susceptibility to viral infection. For instance, BTK-deficient macrophages were incompetent to produce inflammatory cytokines to clear intracellular dengue virus [319].

The emergence of the COVID-19 pandemic has recently urged the development of effective therapeutics. An overactivation of macrophages was closely related to the hyperinflammatory status in severe cases of COVID-19, including those with acute respiratory distress syndrome. The regulatory effects of BTK on macrophages made it promising to repurpose BTKi in managing severe COVID-19 patients. In two pilot studies, CLL and WM patients treated with ibrutinib developed only mild symptoms after severe acute respiratory syndrome coronavirus 2 (SARS-CoV-2) infection, suggesting that BTK inhibition may protect against SARS-CoV-2 virulence [320, 321]. Limited by the small sample size, more persuasive randomized studies are required. In severe COVID-19 patients, administration of acalabrutinib drastically and rapidly improved oxygenation and normalized inflammation of C-reactive protein and IL-6 with no discernable toxicity [307]. In addition to the anti-inflammatory effects, ibrutinib and zanubrutinib may affect the viral entry and replication steps of SARS-CoV-2 infection [305]. BTKi also prevented thromboinflammation in COVID-19 via direct or indirect interactions with platelets [306]. Notwithstanding, considering the complex factors that contribute to severe SARS-CoV-2 infection, multiple interventions targeting distinct inflammatory pathways are necessary to control the disease entirely. The application of BTKi in managing COVID-19 is being assessed in plenty of clinical trials (NCT04497948, NCT04439006, NCT04382586, etc.). The pending clinical data will help us evaluate the benefit–risk ratio of BTKi in COVID-19 patients.

#### Bacterial infection

BTK has diverse functions in the infection of different bacteria at different stages due to the various immune responses. On the one hand, BTK-dependent innate and humoral immunity is essential for controlling certain types of bacteria. Thus, BTK inhibition impairs the anti-bacterial immunity and promotes infection. For instance, inhibiting BTK with ibrutinib ameliorated macrophage- and γδT cell-mediated immune responses against *Mycobacterium tuberculosis* infection [322]. On the other hand, overactivation of BTK can be detrimental by driving infection-induced hyperinflammation. In pneumococcal pneumonia, ibrutinib treatment controlled acute pulmonary inflammation by inhibiting myeloid cell activation and migration [323]. Hidradenitis suppurativa (HS) is a chronic inflammatory skin disease accompanied by an abundant bacterial infection in the lesions, including *Corynebacterium*, *Porphyromonas,* and *Peptoniphilus* species [324]. Proteomic and transcriptomic analysis of skin lesions from 22 HS patients revealed that activation of BTK and SYK pathways is the central signal transduction network in HS, which promoted the activation of B cells and plasma cells, and facilitated the overproduction of inflammatory cytokines including IFN-γ, IL-36, and TNF [325]. A phase 2 clinical study is recruiting volunteers to determine the efficacy and safety of remibrutinib in 200 patients with moderate-to-severe HS (NCT03827798). In summary, BTKi may have both beneficial and detrimental effects on bacterial infection, depending on the species of the microorganism, the degree of inflammatory responses, and the mechanisms of anti-bacterial immunity.

#### Fungal infections

Generally, fungal infection belongs to opportunistic infection that commonly occurs in immunocompromised patients. Activation of BTK pathways in macrophages plays a significant role in the defense against fungal infection through phagocytosis and immune regulation. In agreement, defective BTK signaling in macrophages increased the susceptibility to pulmonary *aspergillosis* infection [326]. Cancer patients receiving BTKi treatment exhibited a higher incidence of invasive *aspergillosis* [60]. Besides macrophages, BTKi can also impair neutrophil and platelet-mediated antifungal activity [327, 328]. For instance, ibrutinib exposure in CLL or lymphoma patients is associated with defects in neutrophil response, resulting in a higher risk of *Aspergillus fumigatus* infection and *Pneumocystis jirovecii* pneumonia [59, 328]. Thus, in patients receiving BTKi, it is critical to keep a close eye on fungal infections to avoid these life-threatening AEs.

### BTK inhibitors in chronic inflammatory disorders

BTK is critical for thrombus formation by activating botrocetin/von Willebrand factor (vWF) and collage/glycoprotein VI-induced platelet activation [329, 330]. Recent studies indicated that BTKi (ibrutinib, acalabrutinib, and tirabrutinib) blocked platelet aggregation and atherosclerotic plaque-induced thrombus formation in humans [331]. Therefore, BTKi hold great promise as antiplatelets in atherosclerosis patients. BTK signaling is also an essential regulator of the pro-inflammatory process in the lungs, which makes disrupting BTK a promising strategy against chronic pulmonary inflammatory diseases, including chronic obstructive pulmonary disease and asthma [332]. A proof-of-concept phase 2 study is ongoing to evaluate the effects of rilzabrutinib in adult patients with moderate-to-severe asthma (NCT05104892). Atopic dermatitis is a chronic inflammatory skin disease with a broad spectrum of clinical manifestations. Currently, three different selective BTKi are under phase 2 exploration for the management of atopic dermatitis, including branebrutinib (NCT05014438), rilzabrutinib (NCT05018806), and PRN473 (NCT04992546).

## Acquired resistance to BTK inhibitors and possible solutions

Although BTKi have revolutionized the treatment landscape of B cell malignancies, primary and secondary resistance cases have been observed in patients receiving the first- and second-generation BTKi, especially ibrutinib, acalabrutinib, and zanubrutinib. The attainment of drug resistance is closely related to shortened survival. The number of previous treatments and the presence of TP53 mutation are useful predictors of patients with high-risk resistance to BTKi [333]. Multiple mechanisms of BTKi resistance have been reported. The most common one is the mutations on BTK, including the binding site (BTK^Cys481^ and BTK^C481R^), gatekeeper (BTK^Thr474^), and the SH2 domain (BTK^Thr316^) [102]. BTK^Cys481^ is closely related to CXCR4 mutations in WM patients resistant to ibrutinib [181]. Besides, mutations of the downstream signaling molecules, such as PLCγ2, CARD11, and BCL10, have also been documented in ibrutinib- or acalabrutinib-resistant patients [189, 334–336]. These mutations caused prolonged and BTK-independent activation of NF-κB, resulting in cancer cell proliferation and migration. In addition, overactivation of compensatory signaling pathways can protect cancer cells from apoptosis, including PI3K/Akt/mTOR and the non-canonical NF-κB pathways [337]. For instance, aberrancy of TNF receptor-associated factor-2 (TRAF2), TRAF3, and baculoviral inhibitor of apoptosis proteins repeat-containing 3 (BIRC3)) can stimulate cancer cell survival by activating the alternative NF-kB pathway via the intermediate signal molecule mitogen-activated protein kinase kinase kinase 14 (MAP3K14) [338]. Variants in TRAF3, TRAF2, BIRC3, and BIR3 were observed in MCL patients who were primarily resistant to ibrutinib [339]. Finally, del(8p) and tumor microenvironment are essential factors that drive resistance to BTKi [337]. The overexpressed integrin β1 in the microenvironment can interact with the activated PI3K pathway to facilitate tumor growth and ibrutinib-resistant in MCL [340].

It is necessary to overcome the resistance to BTKi to improve the survival outcomes. The potential approaches include the following: (1) Third-generation reversible BTKi whose binding sites are not the Cys481 residues or cover both WT and mutant Cys481. Some representatives of these inhibitors include fenebrutinib, pirtobrutinib, nemtabrutinib, vecabrutinib, and HMPL-760. (2) Targeted therapies to prevent the activation of bypassing signaling pathways, including PI3K inhibitors [334], BCL-2 inhibitors [146, 341], SYK and LYN inhibitors [342], HSP90 inhibitors [343], etc. In a multicenter phase 2 clinical study, venetoclax induced durable clinical response and prolonged survival in CLL patients who progressed after ibrutinib treatment [341]. (3) Cellular therapy, including hematopoietic stem cell transplantation (HSCT) and CAR-T cell therapy. In R/R MCL patients who had received BTKi before, KTE-X19 CAR-T cell treatment showed remarkable efficacy with an objective response rate of 93% and a CR rate of 67% [344]. The estimated 12-year PFS and OS were 61% and 83%, respectively, suggesting CAR-T cell therapy as a promising strategy against BTKi resistance in B cell malignancies. (4) Combined therapy. BTKi can be combined with targeted therapy, immunotherapy, and CAR-T therapy for enhanced response. Combined therapy can hopefully induce deep and durable remission and allow for fixed-duration treatment. This eliminated the requirement of continuous treatment with BTKi, thus reducing the possibility of developing drug resistance. Further studies should focus on investigating the underlying mechanism of drug resistance and evaluating the efficacy and safety of the salvage therapies in the clinic. Moreover, for patients resistant to BTKi, detection of the mutated or abnormal molecules or genes is necessary to guide the individualized treatment.

## Conclusions and perspectives

BTKi are novel targeted agents that have a promising future for the treatment of hematological cancers and inflammatory disorders. Ibrutinib is the best-studied BTKi at present, with longer-term observational results and greater clinical and real-world experience. But several head-to-head comparisons have demonstrated that the highly selective BTKi acalabrutinib and zanubrutinib showed non-inferior activity but significantly higher safety profiles than ibrutinib in R/R CLL patients [96, 223]. The approval of BTKi has lowered the involvement of chemotherapy and revolutionized the treatment landscape of B cell malignancies, including CLL, MCL, MZL, WM, and PCNSL. The development of BTKi in inflammatory diseases majorly focused on highly selective second- and third-generation BTKi with fewer side effects because most patients with inflammation have mild symptoms. Although promising, most data are from preclinical and early-phase clinical studies, making it hard to assess the performance of BTKi in inflammatory diseases objectively and accurately at present.

Although BTKi have succeeded extremely in treating B cell cancers, future efforts are still required: (1) Continuous therapy with BTKi facilitated the development of acquired resistance and toxicities. Hence, it is necessary to search for time-limited combination strategies that could induce deep and durable responses. (2) As more selective BTKi become available now, head-to-head randomized clinical studies are necessary to help identify the optimal agent with higher efficacy and better tolerability in various disorders. (3) Treatment-related AEs should be carefully monitored, especially for the life-threatening ones, including opportunistic infections, atrial fibrillation, bleeding, etc. [96]. (4) Drug resistance should be handled to guarantee the efficacy of BTKi. (5) BTKi have not been approved for other indications apart from B cell cancers, mainly due to insufficient clinical data. Therefore, further studies are necessary to broaden the application of BTK, especially for conditions with undesired clinical therapy. Close cooperation between scientific research institutions, pharmaceutical companies, and medical institutions may accelerate the discovery of new drugs and the optimization of current treatment options.

## Data Availability

Not applicable.

## Abbreviations

BTK: Bruton’s tyrosine kinase
XLA: X-linked agammaglobulinemia
BCR: B cell receptor
BTKi: BTK inhibitors
FDA: Food and Drug Administration
R/R: Relapsed and refractory
MCL: Mantle cell lymphoma
TLR: Toll-like receptor
FcR: Fc receptor
SLE: Systemic lupus erythematosus
(R)MS: (Relapsing) multiple sclerosis
CLL: Chronic lymphocytic leukemia
SLL: Small lymphocytic lymphoma
WM: Waldenstrom macroglobulinemia
MZL: Marginal zone lymphoma
DLBCL: Diffuse large B cell lymphoma
FL: Follicular lymphoma
MM: Multiple myeloma
RA: Rheumatoid arthritis
ITP: Idiopathic thrombocytopenic purpura
PIP3: Phosphatidylinositol-3,4,5-trisphosphate
SYK: Spleen tyrosine kinase
GCB‑DLBCL: Germinal center B-cell-like DLBCL
ABC-DLBCL: Activated B cell DLBCL
BLNK: B cell linker protein
PLCγ2: Phospholipase C-γ2
PI3K: Phosphoinositide 3-kinase
PKCβ: Protein kinase Cβ
CARD11: Caspase recruitment domain-containing protein 11
WT: Wild-type
Cys: Cysteine
AEs: Adverse effects
BID: Twice daily
QD: Once daily
PCNSL: Primary central nervous system lymphoma
IL: Interleukin
TNF-α: Tumor necrosis factor α
CNS: Central nervous system
CIA: Collagen-induced arthritis
CSU: Chronic spontaneous urticarial
PD-1: Programmed cell death protein 1
ORR: Overall response rate
CIT: Chemoimmunotherapy
PFS: Progress-free survival
OS: Overall survival
CR: Complete response
TN: Treatment-naïve
(u)MRD: (Undetectable) minimal residual disease
FCR: Fludarabine, cyclophosphamide, and rituximab
BR: Bendamustine and rituximab
MRR: Major response rate
VGPR: Very good partial response
BNS: Bing–Neel syndrome
COVID-19: Coronavirus disease 2019
SARS-CoV-2: Severe acute respiratory syndrome coronavirus 2
HS: Hidradenitis suppurativa
